# A Deep Learning-Based Denoising Method for Airborne Electromagnetic Data

**DOI:** 10.3390/s26217021

**Published:** 2026-11-06

**Authors:** Yuhang Li, Chong Zhang, Wei Huang, Qingtian Lü

**Affiliations:** 1 State Key Laboratory of Deep Earth and Mineral Exploration, Chinese Academy of Geological Sciences, Beijing 100037, China; liyh021016@163.com (Y.L.); lqt@cags.ac.cn (Q.L.); 2 Institute of Geophysical and Geochemical Exploration, Chinese Academy of Geological Sciences, Tianjin 300309, China; huangwei2012511@163.com

**Keywords:** airborne electromagnetic data, transient electromagnetic, denoising, deep learning, signal-to-noise ratio, signal reconstruction

## Abstract

**Highlights:**

**What are the main findings?**

- A WL-CNN-BiLSTM framework combining fixed wavelet preprocessing, multi-scale convolution, skip connections, and bidirectional LSTM modules was developed and evaluated for AEM/TEM data denoising.
- Although its physical-domain per-trace SNR distribution had a poorer lower tail than those of Median filtering and VMD, with a P10 of −3.41 dB and a minimum of −25.45 dB, WL-CNN-BiLSTM achieved a higher aggregate physical *B*_z_-domain SNR of 17.53 dB than PC-BiLSTMNet (reimplementation), Median filtering (validation-selected), and VMD (validation-selected) on the 3000-trace benchmark test subset.
- On an independent 1000-trace blind test, the separately trained layered-earth configuration using training-only scalers achieved a higher median whole-trace physical-domain SNR of 5.91 dB than validation-selected Median filtering (0.39 dB) and VMD (−0.05 dB), although its lower-tail performance was substantially poorer.

**What are the implications of the main findings?**

- Multi-seed ablation and matched *p* = 0 comparisons indicate that late-time fidelity benefits from empirical temporal feature weighting are not established, demonstrating that the primary denoising power stems from multi-scale recurrent fusion rather than heuristic late-time emphasis.
- Comprehensive structural out-of-distribution (OOD) and noise-stress analyses establish rigorous operational boundaries and preprocessing protocols for deep-learning AEM denoisers, providing vital benchmark insights for community reproducibility.

**Abstract:**

Airborne electromagnetic (AEM) measurements are vulnerable to mixed environmental noise, and rapid signal decay hampers weak transient recovery. We propose WL-CNN-BiLSTM, integrating dual-stage wavelet filtering, parallel multi-scale convolutions, residual skip connections, bidirectional recurrent units, and a hybrid reconstruction objective for complex TEM waveforms. On the archived 20,000-trace benchmark’s 3000-trace test subset, it achieved an aggregate physical-domain SNR 17.53 dB (RMSE 1.48 × 10^−10^ T; PCC 0.989), versus 11.49 dB for PC-BiLSTMNet, 11.76 dB for validation-selected Median filtering, and 13.04 dB for VMD; the per-trace SNR P10 was −3.41 dB (minimum −25.45 dB). These archived results characterize stored numerical targets, not independently verified AEM responses. On an independent 1000-trace blind test, a separately trained layered-earth configuration using training-only scalers achieved a median SNR of 5.91 dB versus 0.39 dB (Median) and −0.05 dB (VMD), but poorer lower-tail SNR (P10 −43.93 dB) and a median of NRMSE 7.402 over 101–1001 μs. Proprietary iFTEM-II windows showed waveform changes versus the engineering curve, but unavailable proxy provenance/calibration made this qualitative evidence, not field-accuracy validation. A matched *p* = 0 comparison, three-seed ablations, complete training-only-scaler retraining, and out-of-distribution diagnostics delineate distributional dependencies; temporal feature weighting is an implemented design component, not a demonstrated late-time enhancer. It shows aggregate reconstruction gains within the benchmark and characterizes dataset- and protocol-dependent limitations.

## 1. Introduction

Airborne electromagnetic (AEM) surveying has become one of the most efficient geophysical tools for large-scale subsurface mapping. By transmitting a pulsed electromagnetic field from an aircraft or helicopter and recording the decaying secondary magnetic field (transient electromagnetic, TEM, response), AEM systems can rapidly image electrical conductivity variations at depths ranging from tens to several hundred meters without ground contact [1,2,3]. This non-invasive, high-coverage capability makes AEM particularly valuable for mineral exploration, groundwater assessment, permafrost characterization, environmental contamination mapping, and geological hazard evaluation [4].

However, raw AEM/TEM data are inevitably corrupted by multiple noise sources. These include natural sferics from global lightning activity, cultural electromagnetic interference from power lines and infrastructure, motion-induced noise caused by aircraft dynamics, and instrument white noise [5,6]. Such contaminations are especially problematic at late times, when the signal amplitude becomes weak and carries essential information about deeper geological structures [7]. Without effective denoising, subsequent inversion becomes unstable, leading to distorted resistivity models and unreliable geological interpretations [8].

Over the past two decades, a variety of traditional signal processing techniques have been developed to mitigate TEM noise. Representative approaches include wavelet thresholding [7,9], variational mode decomposition (VMD) combined with wavelet denoising [10], empirical mode decomposition (EMD) [11], singular value decomposition (SVD) [5], adaptive Kalman filtering [12], curvelet transforms [13], and dictionary-learning methods [14]. While these techniques have achieved notable success in suppressing specific noise types, they often require extensive manual parameter tuning, struggle with mixed or non-stationary noise, and frequently attenuate weak late-time signals, resulting in loss of deep subsurface information [7,10,13].

In recent years, deep learning has emerged as a powerful alternative that learns direct mappings from noisy to clean signals without explicit noise modeling. Recent deep-learning approaches include CNN-based airborne TEM inversion [15], wavelet-enhanced Transformer-based TEM denoising [16], U-Net-based semi-airborne TEM denoising [17], PC-BiLSTMNet [18], and deep-learning frameworks for AEM inversion [19]. These studies differ in task definition, acquisition modality, datasets, and evaluation protocols, so their published results do not provide a controlled performance ranking on the benchmark used in the present study. Despite these advances, existing deep-learning approaches still exhibit several limitations: (i) they often separate denoising from inversion, complicating the overall processing pipeline [8,15,16]; (ii) few incorporate physics-informed constraints tailored to the exponential decay characteristics of TEM signals [18,20]; and (iii) generalization to real field data containing complex, site-specific noise remains limited [17,18,19]. In addition, the trade-off between noise suppression and preservation of subtle geological signals continues to spark debate within the geophysical community [13,21].

To address these signal processing challenges, we propose a wavelet-enhanced convolutional neural network–bidirectional long short-term memory (WL-CNN-BiLSTM) framework for AEM data denoising. The framework combines fixed wavelet processing, multi-scale convolutional feature extraction, residual and skip connections, bidirectional LSTM modules, and a hybrid reconstruction objective. A normalized positional feature-weighting module is retained as an implementation choice and examined in the sensitivity and ablation analyses rather than assumed to provide a late-time fidelity benefit. We evaluate its denoising performance using synthetic noisy–clean signal pairs, comparisons with conventional and learning-based methods, and a field-data transfer assessment.

From a physical sensing perspective, this study focuses on the transient magnetic-flux-density signals recorded by airborne electromagnetic receivers. Our objective is to examine noise suppression and signal fidelity at the sensor output processing stage, including waveform agreement and the reconstruction of low-amplitude late-time responses. The contribution therefore lies in signal processing and quality assessment for electromagnetic sensing measurements, rather than in sensor hardware design or the direct evaluation of subsurface inversion and geological interpretation.

## 2. Methodology

This section details the proposed deep learning-based denoising method for AEM data. The approach integrates synthetic data generation using established geophysical simulation tools [22,23], advanced preprocessing techniques, and WL-CNN-BiLSTM architecture. Mature methods, such as wavelet transforms [9] and variational mode decomposition (VMD) [10], are briefly described with appropriate citations, while novel elements, including the multi-scale feature fusion and hybrid loss function, are elaborated in detail. All implementations are based on Python 3.13 with libraries such as PyTorch, SimPEG [24], Discretize, pywt, and vmdpy. The code is structured to run on standard hardware, with optional GPU acceleration for training efficiency.

### 2.1. Overall Workflow

**Figure 1:**
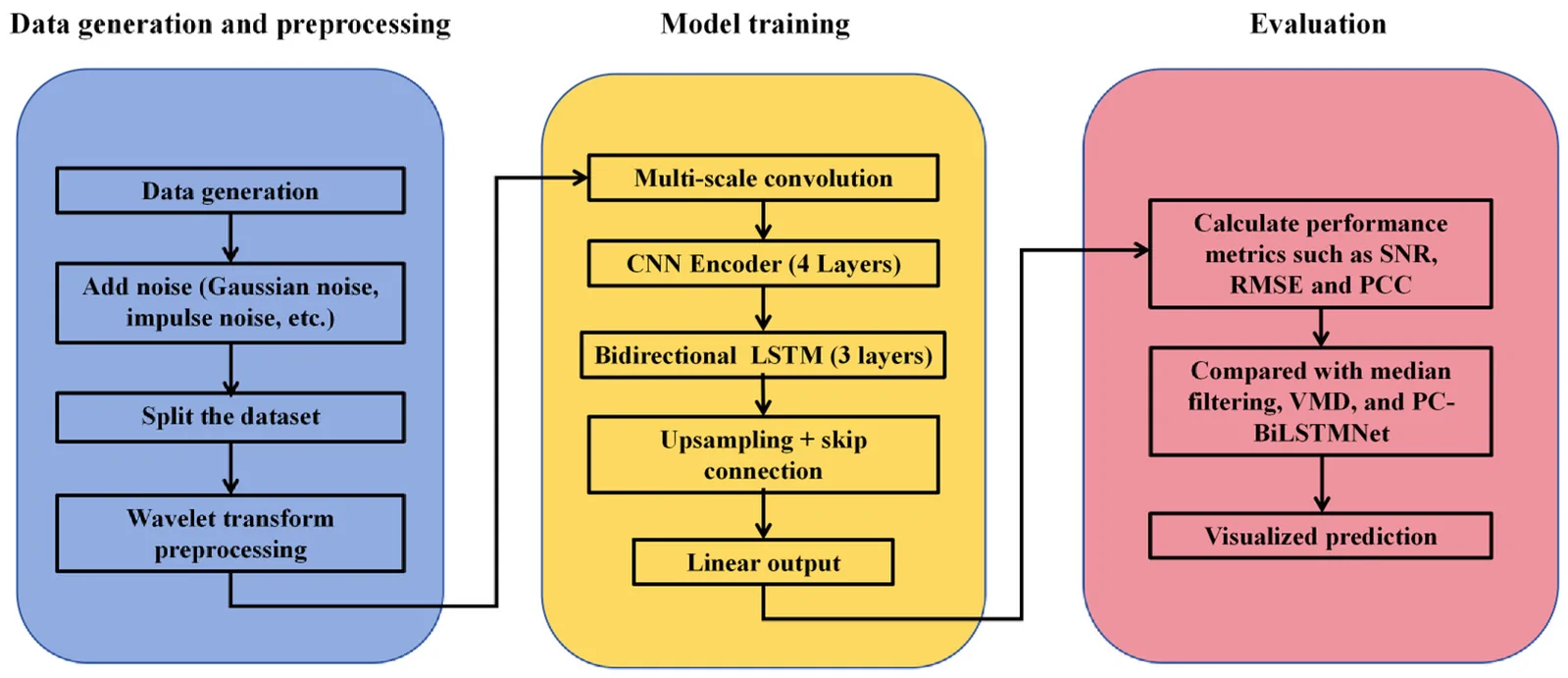
Overall workflow of the proposed denoising method, including data generation and preprocessing (**left**), model training with a hybrid loss (**center**), and benchmark evaluation (**right**). The workflow addresses synthetic mixed noise comprising Gaussian, impulse, and sferic components.

The methodology comprises three main stages: data generation and preprocessing, network training, and denoising application, as illustrated in Figure 1. The workflow begins with synthetic AEM data generation to create a diverse dataset mimicking real-world geological variability and noise conditions. Preprocessing involves wavelet-based preliminary denoising and normalization to prepare the data for model input. The WL-CNN-BiLSTM model is then trained using a hybrid loss function and evaluated against benchmarks like median filtering and VMD. Finally, the trained model is applied to test data for denoising, with performance metrics such as signal-to-noise ratios (SNR), root mean square error (RMSE), and Pearson correlation coefficient (PCC) computed for validation.

We used SimPEG [24] to generate a numerical model-development benchmark from procedurally varied conductivity and thickness parameters. The benchmark contains 20,000 synthetic signal pairs, divided into 70% training, 15% validation, and 15% testing subsets. The parameter-generation procedure, three-dimensional forward implementation, and limitations of the resulting geological interpretation are described in Section 2.2.

### 2.2. Synthetic Data Generation

To provide paired signals for model development, we generated 20,000 synthetic samples using SimPEG [24]. Each realization was initialized as a sequence of thickness and conductivity parameters. The first thickness was sampled uniformly between 20 and 500 m, and subsequent thicknesses were sampled between 300 and 1000 m until their cumulative sum reached at least 2000 m. Each thickness was then multiplied by a random factor between 0.8 and 1.2. The base conductivity values were logarithmically spaced between 10^−5^ and 1 S/m and multiplied by two successive lognormal factors, each generated with a log-space mean of zero and a standard deviation of 0.5. The final number of parameter entries and their cumulative thickness depended on the subsequent insertion and replacement operations.

**Figure 2:**
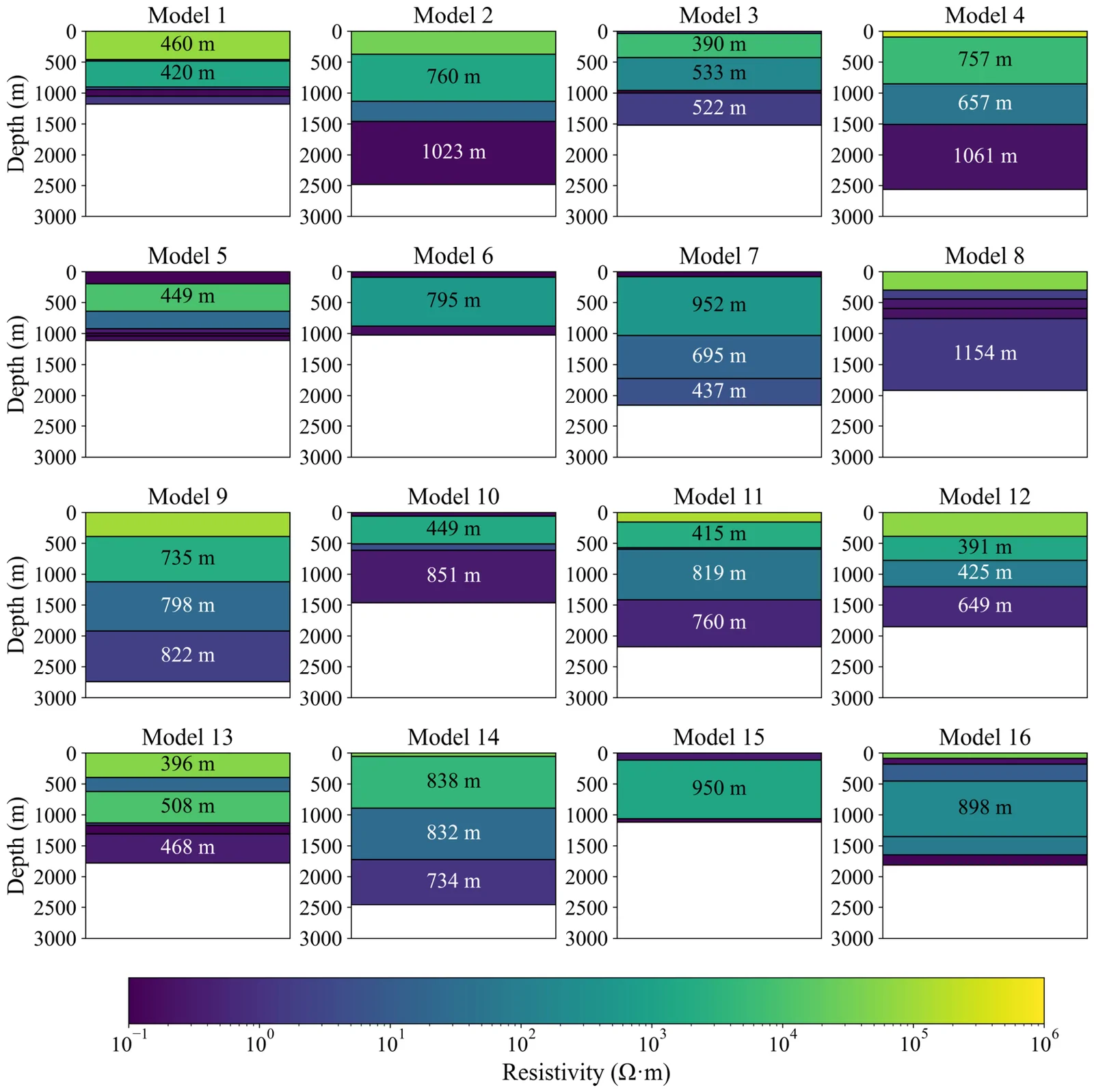
Sixteen illustrative resistivity parameter profiles independently generated using the same procedural rules as the model-development benchmark. The profiles were regenerated for visualization and were not selected from the archived 20,000-pair corpus. All panels use a common depth range of 0–3000 m and a common logarithmic resistivity scale. The profiles are illustrative parameter sequences and should not be interpreted as a direct representation of realistic three-dimensional geological bodies.

We selected one of six procedural variants, labeled “layered,” “faulted,” “intrusive,” “folded,” “volcanic,” and “aquifer” in the generation code. These labels identify alternative rules for modifying the parameter sequence, including conductivity perturbation, index-dependent conductivity modulation, and insertion of an additional thickness–conductivity pair; they do not define separate three-dimensional geological geometries. We then performed zero to five additional replacement operations. In each operation, one parameter entry was selected and its conductivity and thickness were replaced by values sampled uniformly from 0.001–10 S/m and 10–200 m, respectively. The same entry could be selected more than once. These operations modify the parameter lists rather than insert spatially localized blocks with specified horizontal dimensions or boundaries. Figure 2 illustrates 16 independently regenerated parameter profiles using the same generation rules; they are visualization examples rather than samples selected from the archived 20,000-pair corpus. The kilometer-scale cumulative depths of these parameter sequences are part of the numerical model-development benchmark and do not represent an estimate or claim of AEM survey penetration or depth of investigation.

A circular-loop transmitter with a radius of 10 m was centered at (0, 0, 10) m, and the receiver was located at (15, 0, 10) m. The receiver therefore had a horizontal offset of 15 m from the loop center and was not on the transmitter axis. We used SimPEG’s PointMagneticFluxDensity receiver with its orientation set to z to calculate the vertical magnetic flux density *B*_z_ in tesla (T), rather than its time derivative. The excitation was represented by StepOffWaveform, which assumes an instantaneous current shutoff without a finite turn-off ramp. The response was sampled at 101 linearly spaced times from 1 to 1001 μs after shutoff, with a constant interval of 10 μs. The first sample at 1 μs defines the lower time bound of the synthetic benchmark and matches the initial numerical time-step size. It is not intended to represent the earliest usable measurement gate of a particular airborne instrument.

Forward responses were calculated using SimPEG’s Simulation3DMagneticFluxDensity on a three-dimensional tensor mesh [24]. The horizontal grid contained 30 × 30 cells of 5 m × 5 m and a lateral extent of 150 m in each direction. Each thickness entry defined a single vertical cell without internal subdivision; before insertion and replacement operations, the perturbed widths were 16–600 m for the first entry and 240–1200 m for the remaining entries. For comparison, the characteristic diffusion length $l_{d}=\sqrt{2t/\left(\mu_{0}\sigma \right)}$ is 12.62 m at 1 μs for σ=0.01 S/m. Across the base conductivity range of 10^−5^–1 S/m, it spans 1.26–398.94 m at 1 μs and 39.91–12,621.97 m at 1001 μs; at 10 S/m, permitted by conductive insertion and replacement, the corresponding lengths are 0.40 and 12.62 m. These illustrative scales show that the coarse vertical cells cannot resolve early-time diffusion in conductive materials, while the limited lateral extent is inadequate for larger diffusion scales. The implementation lacked a separate low-conductivity air region and boundary-padding cells, and the audit identified parameter-to-cell assignment inconsistencies.

To assess the consequences of this implementation, we generated 100 new parameter sequences using the archived parameter-generation procedure with a fixed random seed of 20261029. Each sequence was evaluated using both the archived tensor-mesh implementation and Simulation1DLayered, with the same nominal transmitter–receiver geometry, step-off excitation, and 101 receiver times. For the one-dimensional reference, all thicknesses except the final entry defined finite layers, and the final conductivity defined an infinite bottom half-space. The circular transmitter was approximated by a closed 128-segment polygon with five integration points per segment. These sequences were generated specifically for the diagnostic; they were not recovered from the original 20,000-trace corpus, whose per-trace parameter correspondence was not retained.

All 100 response pairs were finite. The relative L_2_ discrepancy, defined as the norm of the difference between the three-dimensional and one-dimensional responses divided by the norm of the one-dimensional reference, had a median of 114.7306. Seventeen of the 100 tensor-mesh responses changed sign over the sampled interval, whereas none of the corresponding one-dimensional responses did. Together with the mesh-scale assessment, these results indicate that the archived three-dimensional responses are dominated by discretization and boundary artifacts, compounded by the missing air region and parameter-to-cell assignment errors. The diagnostic does not isolate the contribution of each implementation deficiency. We therefore treat the sign-changing and non-monotonic archived references as numerical artifacts, rather than physical effects attributable to receiver offset. Reconstruction scores against these stored targets quantify agreement with the archived numerical outputs, not recovery of physically verified AEM signals. The separate layered-earth consistency checks described below do not validate or correct the archived tensor-mesh corpus.

The numerical time-step sequence consisted of 100 steps of 1 μs, followed by 100 steps of 5 μs and 100 steps of 10 μs. This was a prescribed time-step schedule rather than adaptive error-controlled stepping. The implementation selected SolverLU when available and otherwise used the solver default. For supervised denoising, the simulated response before the addition of synthetic noise served as the reference signal.

The material-property variations in the forward models were restricted to electrical conductivity. We did not model induced polarization (IP), superparamagnetic responses, or magnetic-permeability contrasts.

We added Gaussian noise, impulse noise, and a sparse-spike component referred to as sferic noise to the simulated reference responses. For each trace, the Gaussian standard deviation was set to a randomly selected fraction between 0.05 and 0.50 of the absolute clean-signal amplitude at each receiver time. The impulse occurrence probability, *q*, was sampled uniformly between 0.10 and 0.30 for each trace, and each sample was independently selected for impulse contamination with that probability. At the selected samples, impulse amplitudes were generated by multiplying independent values drawn uniformly from −1 to 1 by the maximum absolute clean-signal amplitude and a trace-specific scale factor sampled uniformly between 0.5 and 1.5. The sferic component contained one to five randomly located spikes, with amplitudes sampled uniformly between −1.5 and 1.5 times the maximum absolute clean-signal amplitude.

The Gaussian component defines a signal-dependent perturbation whose standard deviation scales with the local reference amplitude; it does not include a signal-independent additive noise floor. Its standard deviation therefore approaches zero as the reference amplitude approaches zero. The impulse and sferic components are instead scaled by the trace peak and can remain large relative to weak portions of the response. This construction defines a controlled synthetic corruption setting rather than a calibrated model of an instrument’s absolute noise floor or detection limit.

The interval *q* = 0.10–0.30 specifies the probability of adding the impulse component, not a fixed realized percentage of contaminated samples in each trace. We used this interval as a deliberately severe synthetic corruption range rather than as a measured estimate of typical field contamination after spike rejection. The noise construction represents selected additive perturbations and is not calibrated to reproduce the complete interference statistics of an airborne survey.

Each generator call created a new Earth model and one corresponding noisy–clean trace pair; multiple noise realizations or augmented copies of the same generated model were not created. The 20,000 pairs were randomly divided into 70% training, 15% validation, and 15% testing subsets using a fixed split seed of 42. Under this generation scheme, trace-level partitioning is equivalent to Earth-model-level partitioning. No exact duplicate clean traces, noisy traces, or noisy–clean pairs were found within or across the three subsets. Because the archived arrays do not retain Earth-model identifiers or the original split-index manifest, strict retrospective model-identity verification is unavailable.

**Figure 3:**
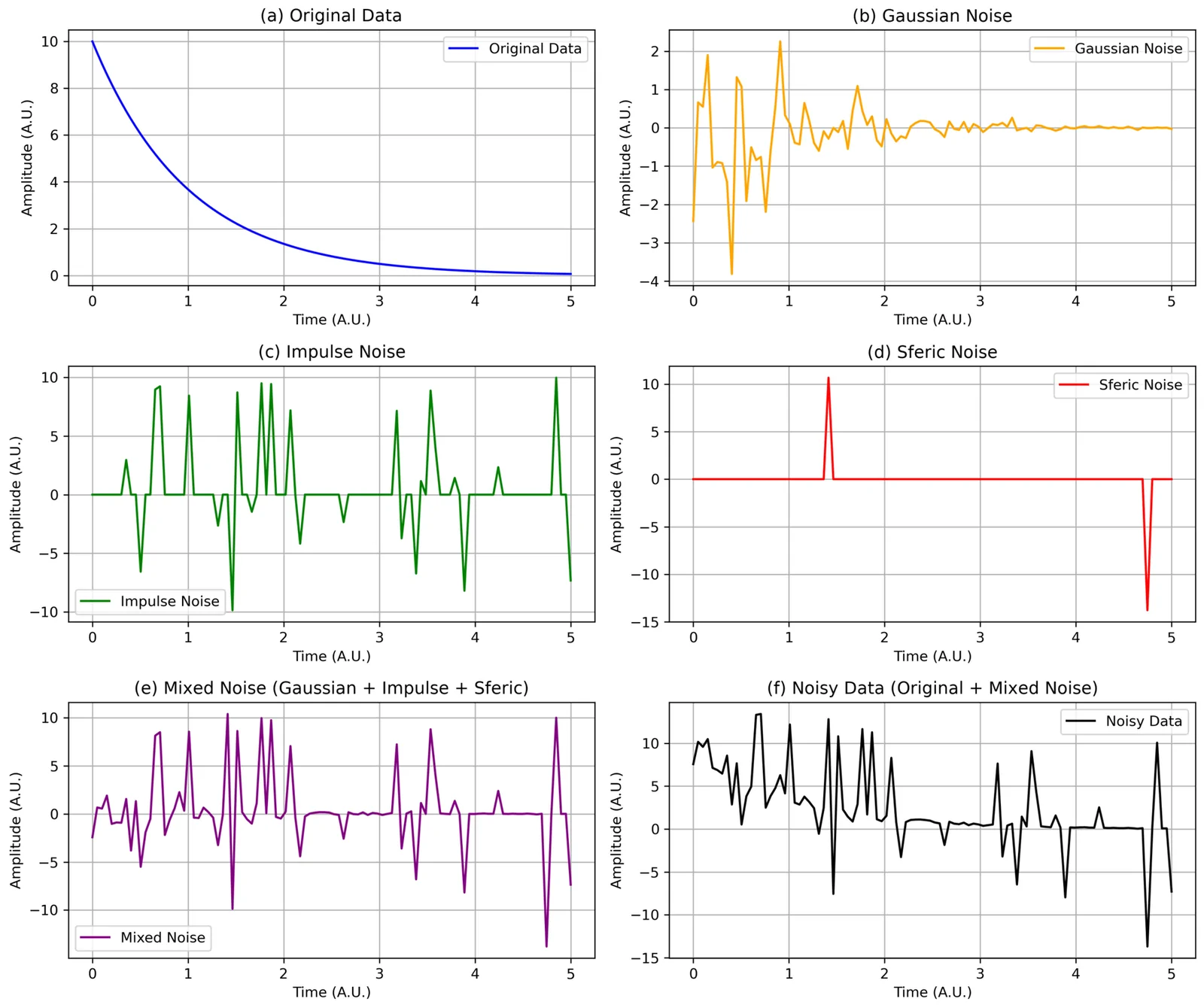
Dimensionless schematic illustration of (**a**) an illustrative decay curve, (**b**) Gaussian noise, (**c**) impulse noise, (**d**) sferic noise, (**e**) the combined noise component, and (**f**) the resulting noisy signal. Time and amplitude are expressed in arbitrary units (A.U.) and do not represent the physical receiver times or magnetic-flux-density amplitudes used in the model-development corpus.

Figure 3 provides a dimensionless schematic illustration of the individual and combined noise components; it is not a simulated AEM record from the model-development corpus.

A separate synthetic dataset was used for the exponent-sensitivity experiment in Section 3.3 and the impulse-sensitivity, structural OOD, and noise-stress experiments in Section 3.5 and Section 3.6. These responses were generated using SimPEG’s Simulation1DLayered solver [24], rather than the tensor-mesh implementation used for the archived 20,000-sample benchmark. Each Earth model consisted of horizontally uniform layers over an infinite bottom half-space. The total number of layers, including the bottom half-space, was sampled uniformly from the integers 3–8. Finite-layer thicknesses were sampled uniformly from 20 to 500 m, and layer conductivities were sampled independently and uniformly in log10 space between 10^−5^ and 1 S/m. These models do not contain lateral geological variations.

The transmitter was a single-turn loop of radius 10 m, carrying 1 A before an ideal step-off, with its center at (0, 0, 10) m. The receiver was located at (15, 0, 10) m and measured *B*_z_ in tesla at the same 101 receiver times from 1 to 1001 μs. For the off-axis receiver geometry, the loop was represented by a closed 256-segment polygon using SimPEG’s LineCurrent source, with five integration points per segment. The calculation used the numerical digital filters key_101_2009 for the Hankel transform and key_81_2009 for the time transform within SimPEG. The layered-earth solver assumes an insulating air region above the ground surface.

We assessed numerical consistency using 12 canonical Earth models covering homogeneous half-spaces, layered conductivity contrasts, and thin conductive and resistive layers. The one-dimensional responses were compared with SimPEG’s Simulation3DMagneticFluxDensity solutions on a refined axisymmetric cylindrical mesh under the same source geometry, waveform, and receiver times. The PDE calculation represented air with a conductivity of 10^−8^ S/m. Across the 12 cases, the maximum reconstruction-difference norm normalized by the one-dimensional reference norm was 2.56%, and the maximum 95th-percentile gatewise relative difference was 5.75%. Gatewise relative differences were evaluated where the reference amplitude was at least 10^−6^ of its peak magnitude. These results satisfied the prescribed limits of 5% and 10%, respectively. Additional checks examined polygon discretization, integration quadrature, transform-filter sensitivity, and PDE discretization. Here, “numerically checked” refers to these numerical consistency checks for the stated layered-earth setting; it does not imply validation against field measurements or establish accuracy for arbitrary three-dimensional geology.

The separate WL-CNN-BiLSTM configuration was trained from random initialization using 8000 training traces and 1500 validation traces generated with this layered-earth procedure. Training noise followed the Gaussian, impulse, and sferic definitions given above. The input and target scalers were fitted only to the training subset. The exponent-sensitivity experiment compares *p* = 0, 1.0, 1.5, and 2.0 within this training–validation configuration. The *p* = 0 and *p* = 1.5 models were evaluated as a matched comparison using the same data split and paired random seed, whereas the *p* = 1.0 and *p* = 2.0 entries are the previously completed single-run sensitivity cases. Section 3.5 and Section 3.6 use the frozen *p* = 1.5 checkpoint and its associated scalers, which was prespecified for those subsequent tests and was not selected as the optimal exponent from the results reported here. These experiments are separate from the archived-benchmark method-comparison and ablation runs described in Section 3.2 and Section 3.3.

### 2.3. Data Preprocessing

Prior to network input, we applied wavelet thresholding followed by Min–Max normalization. For the wavelet preprocessing step, each 101-sample noisy trace was decomposed independently using a three-level Daubechies-4 (db4) discrete wavelet transform (DWT) [7]. Periodization extension was used for both decomposition and reconstruction. The decomposition produced one level-three approximation subband and three detail subbands, denoted by *cA*_3_, *cD*_3_, *cD*_2_, and *cD*_1_, respectively. The first-level detail subband, *cD*_1_, supplied the coefficient statistic used to estimate the noise scale.

For each trace, we calculated a common soft threshold using a scaled universal-threshold expression [9]:

(1) $$\lambda =0.8\hat{\sigma }\sqrt{2lnN},\;\hat{\sigma }=\frac{{median}_{k}\left(\left|d_{1,k}\right|\right)}{0.6745},$$

where λ denotes the soft-threshold value, $\hat{\sigma }$ is the coefficient-based noise-scale estimate, $d_{1,k}$ is the *k*-th coefficient in the first-level detail subband *cD*_1_, and *N* = 101 is the number of samples in the input trace. The operator ${median}_{k}$ takes the median over the coefficients in *cD*_1_, and ln denotes the natural logarithm. The constant 0.6745 rescales the coefficient statistic, while 0.8 is the fixed multiplier applied to the universal-threshold expression. The threshold has the same amplitude units as the corresponding wavelet coefficients. The estimate uses the median of the absolute detail coefficients without first subtracting their median.

The threshold was calculated separately for each trace and applied to all three detail subbands; it was not re-estimated independently at each decomposition level. The approximation coefficients *cA*_3_ were retained without thresholding. The signal was then reconstructed by the inverse DWT using periodization extension and truncated to the input length of 101 samples. Because the record is short, coefficients near its boundaries, particularly at the coarser decomposition levels, can be influenced by the extension procedure.

Subsequently, for the archived model-development benchmark, separate global Min–Max transformations were applied to the wavelet-preprocessed noisy inputs and the clean targets:

(2) $$x^{\prime }=\frac{x-x_{min}}{x_{max}-x_{min}}.$$

Here, $x_{min}$ and $x_{max}$ are the corpus-level extrema of the corresponding scaler. Each scaler was fitted to the flattened 20,000 × 101 amplitudes before the train/validation/test split and was then shared across all traces and receiver-time positions; the transformation was therefore not fitted independently to each trace. Consequently, the corpus-wide extrema map to 0 and 1, whereas an individual trace need not span the full interval. Because validation and test amplitudes contributed to the fitted extrema, this historical preprocessing introduces information leakage and is retained only to reproduce the archived model-development benchmark. The separate layered-earth exponent-sensitivity and stress experiments instead used scalers fitted only to their training subset.

This step ensures consistent input magnitude for the neural network [25]. For the model-development benchmark, we used the precomputed, preprocessed noisy–clean pairs without additional random time shifts, receiver-time jitter, or multiplicative amplitude-scaling augmentation during training. The data loader randomized the order of trace pairs between epochs but preserved the ordering of the 101 receiver-time samples within each trace and the correspondence between each noisy input and its clean target. Each sequence position therefore retained its association with the receiver times specified in Section 2.2. Variability across the training examples arose from the parameter generation and additive noise construction described in Section 2.2, rather than from on-the-fly transformations of the stored traces.

### 2.4. Network Architecture (WL-CNN-BiLSTM)

The WL-CNN-BiLSTM model is specifically designed for AEM denoising. It processes input sequences of length 101 and combines the strengths of convolutional layers for multi-scale feature extraction with recurrent layers for temporal dependency modeling [26].

The normalized input tensor has dimensions *n_b_* × 1 × 101, where *n_b_* denotes the batch size. Before convolutional feature extraction, each sequence passes through a second, fixed wavelet-thresholding stage implemented within the network. This stage uses a three-level db4 decomposition with the default symmetric extension in PyWavelets. The threshold in Equation (1) is recalculated from the first-level detail coefficients of the normalized input and applied to all three detail subbands, while the approximation coefficients remain unchanged. Reconstruction uses the same symmetric extension and is truncated to 101 samples. This internal stage is distinct from the periodization-based preprocessing applied before normalization. It contains no trainable parameters and is implemented outside the automatic-differentiation graph; the subsequent convolutional and recurrent modules are trainable.

The two stages were included to provide preliminary noise suppression before normalization and an additional fixed thresholding operation on the normalized sequence before learned feature extraction. The internal threshold is recalculated from its input rather than reused from the external stage. We retain the external periodization and internal symmetric extensions to preserve the preprocessing and network configuration used for training and evaluation of the reported models. The ablation in Section 3.3 examines the inclusion of the internal stage while retaining the external preprocessing. No matched ablation removing the external stage from WL-CNN-BiLSTM or controlled comparison of boundary-extension combinations is reported. The results therefore characterize the implemented pipeline without establishing that two stages are generally necessary or that this combination of extension modes is optimal.

Multi-scale feature fusion is achieved via three parallel 1D convolutions (kernel sizes 3, 5, and 7; 32 channels each), which are concatenated to yield 96 channels, followed by batch normalization (momentum 0.1), ReLU activation, and dropout (0.15) [27]. This design captures noise patterns at different temporal scales, inspired by the multi-scale convolution of the Inception architecture [27].

The encoder receives the 96-channel fused features and comprises four one-dimensional convolutional blocks with output channel counts of 32, 64, 128, and 256 and kernel sizes of 3, 5, 7, and 3, respectively. Each convolution is followed by batch normalization and ReLU activation, including the fourth and final encoder convolution. Dropout with a rate of 0.15 is applied after ReLU in the first, second, and fourth blocks, but not in the third block. A single max-pooling operation with a kernel size of 2 and ceiling-mode rounding is applied after the fourth block, reducing the sequence length from 101 to 51. A residual branch projects the internally wavelet-processed input to 256 channels using a 1 × 1 convolution with stride 2, and its output is added to the pooled encoder features.

In parallel, a three-layer bidirectional LSTM with 128 hidden units per direction processes the internally wavelet-processed sequence. The standard PyTorch LSTM dropout parameter was set to 0.15; thus, dropout acts only between stacked LSTM layers during training, rather than on recurrent hidden- or cell-state transitions. No additional recurrent or variational dropout was implemented.

The decoder employs a transposed convolution for up-sampling, concatenates the resized deepest encoder features, and applies a second three-layer bidirectional LSTM with 128 hidden units per direction using the same inter-layer dropout rate of 0.15.

After decoder recurrent processing, the feature sequence is truncated to 101 positions and multiplied by the normalized temporal feature weights. A final affine projection with 256 input features and one output, implemented as a linear layer with a bias term, then produces one predicted amplitude in the normalized clean-target domain at each sequence position. No additional ReLU, sigmoid, tanh, or clipping operation is applied to the projected output. The ReLU in the final encoder block therefore acts on intermediate features rather than imposing non-negativity on the predicted signal. The predicted amplitudes are not constrained by an output activation to be nonnegative or to lie within [0, 1]. Physical *B_z_* amplitudes are recovered using the inverse clean-target transformation.

All convolutional and transposed convolutional weights are initialized using the Kaiming method to ensure stable training with ReLU activations [28].

**Figure 4:**
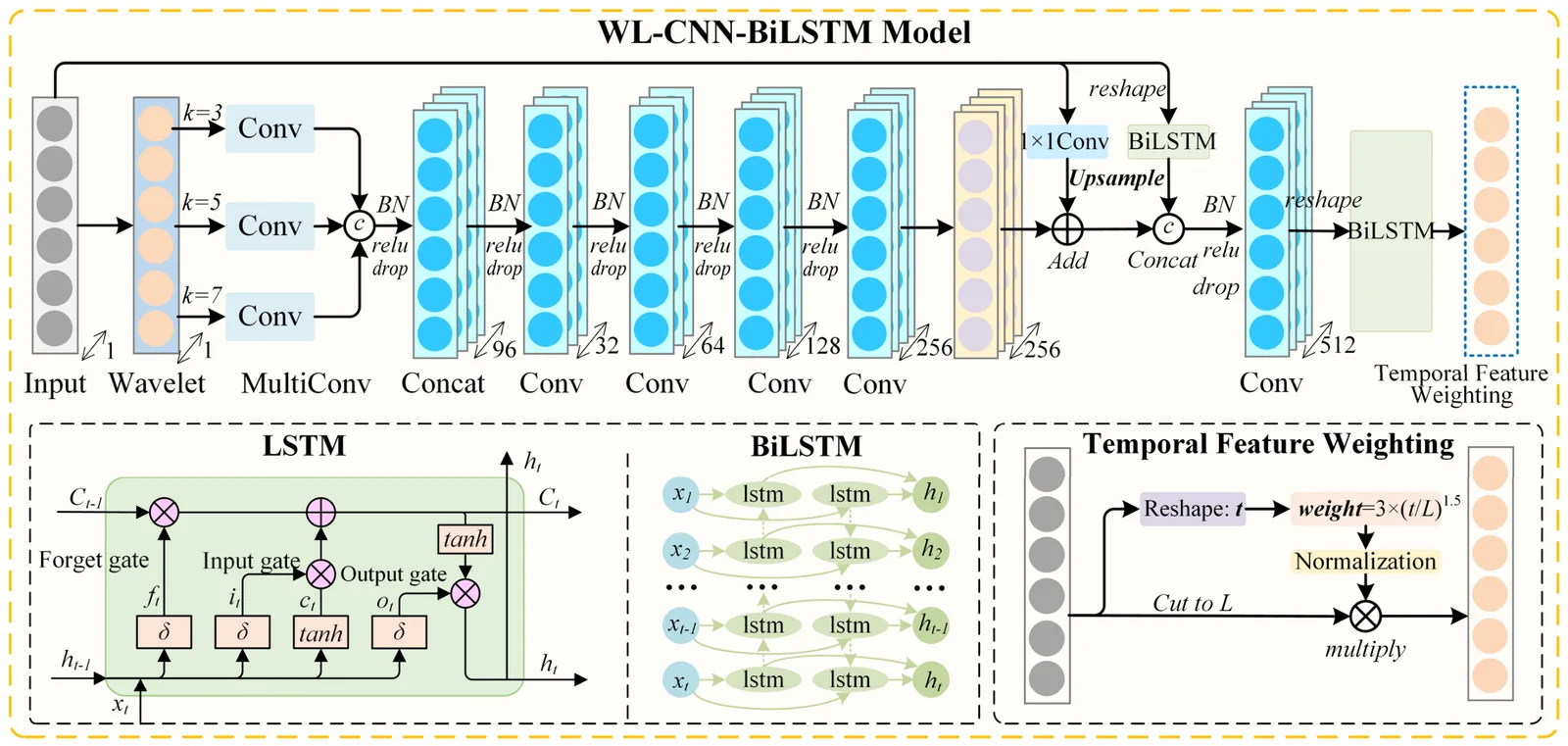
Detailed architecture of the WL-CNN-BiLSTM model, illustrating multi-scale fusion, encoder–decoder structure with skip connections, parallel LSTM branch, and temporal feature weighting.

The overall architecture (Figure 4) combines skip connections [29], multi-scale feature fusion, recurrent processing, and temporal feature weighting for AEM signal reconstruction. Whether these components improve late-time fidelity must be assessed using late-time reconstruction metrics rather than inferred from the architecture alone.

### 2.5. Loss Function and Training Details

WL-CNN-BiLSTM was trained with a fixed combination of amplitude-error, SNR, and correlation terms in the normalized clean-target domain:

(3) $$L=0.5\cdot L_{MSE}+0.15\cdot L_{MAE}+0.1\cdot L_{SNR}+0.15\cdot L_{PCC},$$

where $L_{MSE}=M=\left\langle {\left(\hat{y}-y\right)}^{2}\right\rangle$ and $L_{MAE}=\langle \left|\hat{y}-y\right|\rangle$ are the mean squared error and mean absolute error, respectively. Here, y and $\hat{y}$ denote the clean target and model prediction in the normalized clean-target domain, and the averages are taken over all traces and all 101 receiver-time samples within a batch. Defining the target power as $P=\langle y^{2}\rangle$, the implemented SNR loss is $L_{SNR}=clamp\left(10{log}_{10}\left[\left(M+10^{-10}\right)/P\right],-200,200\right)$. This training quantity is calculated from batch-aggregated powers, rather than by averaging per-trace SNRs. The correlation term is $L_{PCC}=0.1\left[1-clamp\left(\rho ,0,1\right)\right]$, where *ρ* is calculated after flattening the predictions and targets across the batch and receiver-time dimensions [15,18].

The outer coefficients 0.5, 0.15, 0.1, and 0.15 were used as fixed relative weights and were not normalized to sum to one. Because $L_{PCC}$ includes an internal factor of 0.1, the effective coefficient on $1-clamp\left(\rho ,0,1\right)$ is 0.015. These coefficients do not imply equal contributions to the training gradient. For positive target power and an SNR loss strictly within the unclipped interval, the gradients with respect to the predictions satisfy $\nabla_{\hat{y}}\left(0.1L_{SNR}\right)=\frac{2}{ln\left(10\right)\left(M+10^{-10}\right)}\nabla_{\hat{y}}\left(0.5L_{MSE}\right)$.

Thus, the SNR term supplies an error-dependent rescaling of the MSE gradient in the same direction, and its contribution can exceed that of the MSE term by orders of magnitude when the normalized batch MSE is small. In the saturated regions of the SNR clamp, its gradient contribution is zero. The implemented objective therefore combines terms with different effective gradient scales; no adaptive gradient-balancing procedure was applied. Component-wise gradient histories were not recorded, so the relation above describes an analytical property of the implemented loss rather than a measured account of the relative contributions throughout training.

The temporal feature weights are defined as

$$u_{i}=3{\left(\frac{i}{L}\right)}^{p},\;w_{i}=\frac{u_{i}}{L^{-1}\sum_{j=1}^{L}u_{j}},\;i=1,\ldots ,L,$$

where i is the sequence-position index, *L* = 101 is the sequence length, $u_{i}$ is the unnormalized positional weight, $w_{i}$ is the corresponding unit-mean normalized weight, and *p* is the power-law exponent. The exponent *p* controls the shape of the positional weighting and determines how strongly the relative feature emphasis increases toward later sequence positions. For the archived model-development configuration, *p* was fixed at 1.5. This value was used as a heuristic design setting; it was not derived from a TEM physical law, adopted as a calibrated physical parameter, or selected through a formal hyperparameter optimization. Therefore, the main benchmark results should not be interpreted as demonstrating that *p* = 1.5 is optimal.

The *p* = 0 setting produces unit normalized weights and therefore represents the corresponding unweighted reference. The weighting module is retained here to document the evaluated implementation, but the present experiments do not establish it as a beneficial mechanism for preserving weak late-time signals.

Because the normalized weights are divided by their mean, the multiplicative factor 3 cancels exactly during normalization and has no effect on the implemented normalized weights. It is therefore not an independent tuning parameter. The normalized weights multiply the decoder features immediately before the final linear projection and do not implement a gate-wise weighted MSE.

We examined sensitivity to the temporal feature-weighting exponent using *p* = 0, 1.0, 1.5, and 2.0 in the separate layered-earth configuration described in Section 2.2. The *p* = 0 and *p* = 1.5 configurations were evaluated as a matched comparison using the same training–validation split, paired random seed, training-fitted input and target scalers, and validation-based checkpoint-selection procedure. The *p* = 1.0 and *p* = 2.0 entries were retained from the previously completed single-run sensitivity experiment. The test subset was not used in this analysis. The resulting comparison is therefore interpreted as evidence about the tested configurations rather than as a multi-seed estimate of the optimal exponent.

We trained WL-CNN-BiLSTM using Adam with decoupled weight decay (AdamW), with an initial learning rate of 0.0003 and a weight-decay coefficient of 5 × 10^−4^ [30,31]. The batch size was 256, and training was limited to 800 epochs. Two learning-rate schedulers operated on the same optimizer: cosine annealing (CosineAnnealingLR) and learning-rate reduction on a validation plateau (ReduceLROnPlateau). We initialized the cosine scheduler once before training, with T_max_ equal to the number of training batches per epoch and a minimum learning rate of zero. After each training batch, we first updated the model parameters and then advanced the cosine scheduler by one step. Its step counter continued across epochs without reinitialization. Thus, T_max_ was measured in batch updates, and the learning rate could increase again after reaching the cosine minimum; the schedule was not a single monotonic decay over the full training run.

At the end of each epoch, we evaluated the model on the validation subset in evaluation mode without gradient computation. The validation score was the arithmetic mean of the batchwise mean squared errors in the normalized clean-target domain, with each batch error averaged over its traces and receiver-time samples. We then passed this score to ReduceLROnPlateau, configured with mode = ‘min’, factor = 0.1, and patience = 5. The remaining settings retained their defaults: a relative improvement threshold of 10^−4^, zero cooldown, a minimum learning rate of zero, and a minimum learning-rate change of 10^−8^.

When the number of consecutive validation checks without sufficient improvement exceeded five, the plateau scheduler attempted to multiply the optimizer’s current learning rate by 0.1, subject to its minimum-change criterion. This operation did not reset the cosine step counter or modify the cosine scheduler’s stored base learning rate. Subsequent batch updates continued the cosine recurrence using the shared optimizer learning rate. Checkpoint selection and early stopping used the validation MSE as described below. A checkpoint was saved whenever the validation score was strictly lower than the best value previously observed in that training run. An improvement reset the early-stopping counter; otherwise, the counter increased by one. Training stopped after 80 consecutive epochs without a new best validation score or upon reaching the epoch limit. The saved validation-selected checkpoint was reloaded before test evaluation, rather than automatically using the weights from the final training epoch. Within the implemented training routines, test-set metrics did not enter the checkpoint-saving, early-stopping, or learning-rate decisions.

For the model-development benchmark, we did not use test-set SNR, RMSE, or reconstructed test curves to select or adjust the network architecture, learning rates, loss-term coefficients, temporal weighting exponent, or random seeds, or to select among training runs.

All experiments were conducted on an NVIDIA RTX 5070 GPU using PyTorch 2.9.1 [32]. This hardware and software information is provided for reproducibility and does not constitute evidence of model generalization.

## 3. Results and Analysis

This section evaluates the performance of the proposed WL-CNN-BiLSTM model on both synthetic and real AEM data. Quantitative metrics (SNR, RMSE, and PCC) and qualitative visual comparisons are presented. We report a three-seed repeated ablation comparison in which individual components were disabled or the hybrid training objective was replaced with MSE. We also report a matched *p* = 0 versus *p* = 1.5 comparison for the separate layered-earth configuration. The repeated-seed results are summarized using means and sample standard deviations, while paired differences are calculated between each ablation variant and the full model within the same seed. All experiments were implemented in PyTorch 2.9.1 on an NVIDIA RTX 5070 GPU.

### 3.1. Experimental Setup

Synthetic AEM data were generated using SimPEG [24] as described in Section 2.2, producing 20,000 samples with mixed noise (Gaussian, impulse, and sferic). The dataset was split into 70% training, 15% validation, and 15% testing sets. The WL-CNN-BiLSTM model was trained for up to 800 epochs with early stopping (patience = 80) using the hybrid loss function in Equation (3) and AdamW optimizer (initial lr = 0.0003, weight decay = 5 × 10^−4^) [30].

We implemented a PC-BiLSTMNet comparator in PyTorch based on the model description of Cheng and Wu [18], rather than using code or pretrained weights supplied by those authors. Throughout this study, “PC-BiLSTMNet” denotes this local reimplementation. It comprises three parallel convolutional branches with kernel sizes of 3, 5, and 7, branch-level and fused-feature multi-head attention with eight heads, and a two-layer bidirectional LSTM with 32 hidden units per direction. A linear layer maps the concatenated 64-dimensional bidirectional features to a scalar at each recurrent sequence position, and the resulting sequence is linearly interpolated to 101 samples. The hidden size of 32 is the setting used in our implementation, not a value whose agreement with the published configuration has been independently verified.

For the model-development benchmark, both neural models used the same stored training, validation, and test subsets and the same externally preprocessed input arrays. Training used a batch size of 256, a maximum of 800 epochs, and early stopping with a patience of 80 epochs based on validation MSE. However, their optimization settings were not identical. PC-BiLSTMNet used AdamW with an initial learning rate of 10^−4^ and weight decay of 10^−3^, whereas WL-CNN-BiLSTM used 3 × 10^−4^ and 5 × 10^−4^, respectively. The PC-BiLSTMNet training objective combined MSE, MAE, SNR, and correlation terms with coefficients different from those used for WL-CNN-BiLSTM.

Reproduction of the performance reported by Cheng and Wu [18] on their own benchmark has not been established for this implementation. The comparison therefore evaluates the two implemented processing and training configurations within our benchmark; it does not constitute a verified reproduction of the published PC-BiLSTMNet results or a controlled comparison that isolates architecture alone. Median filtering and VMD were also included as conventional comparators, with their validation-selected configurations described below.

We did not include additional published deep-learning methods [16,17,19] as quantitative baselines in the present benchmark. Verified reference implementations, pretrained weights, or original benchmark artifacts required for a controlled reproduction of [16,17] were not available within the present study, while [19] addresses AEM inversion rather than the same denoising task. Constructing simplified surrogate architectures from high-level descriptions would introduce additional implementation choices and would not constitute verified reproductions of the published methods. Therefore, the learning-based quantitative comparison in this study is limited to the local PC-BiLSTMNet reimplementation, and the reported results should not be interpreted as a performance ranking against the broader recent literature.

We quantified synthetic-data reconstruction using signal-to-noise ratio (SNR), root mean square error (RMSE), and Pearson correlation coefficient (PCC). Let $y_{k}$ denote a clean reference amplitude and ${\hat{y}}_{k}$ the corresponding reconstructed amplitude, where *k* = 1, …, *P* indexes the amplitudes included in a particular calculation and *P* is their total number. The reference and reconstruction are always evaluated in the same amplitude coordinate system. For a single complete trace, *P* = *N* = 101, where *N* is the number of receiver-time samples. For the aggregate model-development test metrics, the *M* = 3000 test traces are pooled, giving *P* = *MN* = 303,000, where *M* denotes the number of evaluated traces.

SNR is calculated as

(4) $$SNR=10{log}_{10}\left[\frac{\sum_{k=1}^{P}y_{k}^{2}}{\sum_{k=1}^{P}{\left({\hat{y}}_{k}-y_{k}\right)}^{2}}\right].$$

Here, ${log}_{10}$ is the base-10 logarithm, the numerator is the clean-reference energy, and the denominator is the reconstruction-error energy over the same *P* amplitudes. Dividing both sums by *P* gives the equivalent ratio of mean squared amplitudes. Neither the reference nor the reconstruction error is mean-centered for this calculation. SNR is reported in decibels (dB). The reconstruction error includes both residual noise and signal distortion; the reported output SNR is not an SNR improvement relative to the noisy input.

RMSE is calculated as

(5) $$RMSE=\sqrt{\frac{1}{P}\sum_{k=1}^{P}{\left({\hat{y}}_{k}-y_{k}\right)}^{2}}.$$

In Equation (5), ${\hat{y}}_{k}-y_{k}$ is the amplitude error at the *k*-th evaluated sample, and the factor 1/*P* averages its square over all included samples. RMSE has the same units as the evaluated amplitudes: it is dimensionless in the normalized target domain and is expressed in tesla (T) in the physical *B_z_* domain.

PCC is calculated as

(6) $$PCC=\frac{\sum_{k=1}^{P}\left(y_{k}-\bar{y}\right)\left({\hat{y}}_{k}-\bar{\hat{y}}\right)}{\sqrt{\sum_{k=1}^{P}{\left(y_{k}-\bar{y}\right)}^{2}}\sqrt{\sum_{k=1}^{P}{\left({\hat{y}}_{k}-\bar{\hat{y}}\right)}^{2}}}.$$

Here, $\bar{y}=P^{-1}\sum_{k=1}^{P}y_{k}$ and $\bar{\hat{y}}=P^{-1}\sum_{k=1}^{P}{\hat{y}}_{k}$ are the mean reference and reconstructed amplitudes, respectively, over the same evaluation vector. For aggregate PCC, these are means over all pooled traces and receiver times; for per-trace PCC, they are calculated separately within each trace. PCC is dimensionless and measures centered linear association. A value close to one does not, by itself, establish accurate amplitude recovery. PCC is undefined when either centered vector has zero norm.

For aggregate reporting, Equations (4)–(6) are applied once to the pooled reference and reconstruction vectors. These values are not the arithmetic means or medians of the corresponding per-trace metrics. For distributional reporting, the equations are first applied separately to each trace, and the resulting metric values are then summarized across traces. Undefined or non-finite values are recorded separately rather than replaced with favorable finite scores. These evaluation quantities are distinct from the regularized and weighted terms used in the training objective.

We additionally use normalized RMSE (NRMSE) for physical-domain relative-error diagnostics:

(7) $$NRMSE=\sqrt{\frac{\sum_{k=1}^{P}{\left({\hat{y}}_{k}-y_{k}\right)}^{2}}{\sum_{k=1}^{P}y_{k}^{2}}}=\frac{RMSE}{\sqrt{P^{-1}\sum_{k=1}^{P}y_{k}^{2}}}.$$

In Equation (7), the denominator normalizes the reconstruction error by the clean-reference amplitude, not by its range or maximum. NRMSE is dimensionless and is reported as a ratio rather than a percentage. For whole-trace NRMSE, all 101 receiver-time samples are included. For interval-restricted evaluation, we use the fixed 101–1001 μs evaluation window, which contains 91 of the 101 linearly spaced receiver times (*P* = 91) and excludes the first ten samples at 1–91 μs. This window covers most of the recorded response and does not by itself identify a physical late-time regime. We therefore refer to the resulting metric as NRMSE over 101–1001 μs. Both the reconstruction-error norm and the clean-reference norm are calculated over this same window. Consequently, a very weak reference over the window can yield a large relative error even when the absolute error is small.

For the model-development benchmark, the common normalized clean-target coordinates are related to the physical magnetic-flux-density coordinates by the retained affine transformation

(8) $$z_{k}=a_{c}B_{k}+b_{c},\;{\hat{z}}_{k}=a_{c}{\hat{B}}_{k}+b_{c}.$$

Here, $B_{k}$ and ${\hat{B}}_{k}$ denote the clean and reconstructed vertical magnetic flux density in T, respectively; $z_{k}$ and ${\hat{z}}_{k}$ are their dimensionless clean-target-scaled values; $a_{c}$ > 0 is the retained clean-target scale factor, with units of T^−1^; and $b_{c}$ is its dimensionless offset. Both coefficients are fixed across the traces and receiver times in this benchmark and are not fitted separately for each test trace. For normalized-domain evaluation, $y_{k}=z_{k}$ and ${\hat{y}}_{k}={\hat{z}}_{k}$; for physical-domain evaluation, $y_{k}=B_{k}$ and ${\hat{y}}_{k}={\hat{B}}_{k}$.

The aggregate results in Section 3.2 report both coordinate representations of the same model-development test outputs. The nonzero offset bc changes the clean-reference energy in Equation (4), so normalized-domain SNR and physical-domain SNR are not interchangeable. RMSE changes with the amplitude scale, whereas PCC is unchanged by the common positive affine transformation, apart from numerical rounding. The separate layered-earth exponent-sensitivity and stress experiments use per-trace metrics in the physical *B*_z_ domain. Comparisons with an engineering proxy for field data quantify agreement with that proxy and are not interpreted as clean-reference SNR measurements. Accordingly, the aggregate normalized-domain metrics are used only as benchmark-level reconstruction summaries and are not taken as direct evidence of late-time signal preservation; per-trace variability and reconstruction errors over 101–1001 μs are assessed separately using physical-domain diagnostics. For the 20,000-sample model-development benchmark, we selected the Median filtering and VMD configurations using the 3000-trace validation subset. The search evaluated 185 candidate settings, comprising five Median kernel widths and 60 VMD parameter combinations, each assessed with three reconstruction rules. Selection maximized aggregate SNR in the common normalized clean-target domain. Ties were resolved using lower RMSE, higher PCC, and a deterministic preference for simpler configurations. The test subset was not used to rank or select these baseline configurations. The selected settings are summarized in Table 1.

The quantitative comparisons in Table 2 and Table 3 and their corresponding distribution and waveform plots use the stored wavelet-preprocessed benchmark inputs. For Median filtering and VMD, we first inverted the noisy-input scaler to restore physical amplitude coordinates, then processed each 101-point trace independently. The resulting physical outputs were transformed with the clean-target scaler for normalized-domain evaluation and retained directly for physical-domain evaluation and plotting. Neural-network outputs were already represented in normalized clean-target coordinates; their physical amplitudes were recovered using the inverse clean-target transformation. The clean reference was restored using the same clean-target inverse. Independent traces were not concatenated before filtering, and the external wavelet preprocessing was not reapplied to the stored inputs.

Median filtering used zero padding at the trace boundaries. Before VMD, each physical-coordinate input was divided by its own maximum absolute amplitude, without subtracting an offset, and the corresponding amplitude scale was restored after reconstruction. The 101-point input was extended to 102 points by repeating its last value and trimmed back to 101 points after processing. The stopping tolerance of 10^−7^ was applied to the peak-scaled representation. This scaling convention was fixed before parameter selection and makes the effective physical-amplitude stopping tolerance depend on the input peak.

Validation selected an 11-point Median kernel and a VMD configuration with *K* = 2, *α* = 2000, and *τ* = 0, where *K* is the number of modes, *α* is the bandwidth-penalty parameter, and *τ* is the dual-ascent step parameter. The selected VMD reconstruction omitted the last returned mode and summed the remaining modes. This is an empirically selected reconstruction rule, not an assumption that the last returned mode always has the highest center frequency or contains only noise.

### 3.2. Synthetic Data Experimental Results

Table 2 summarizes the aggregate reconstruction metrics on the 3000-trace model-development test subset using the evaluation protocol defined in Section 3.1. In the common normalized clean-target domain, WL-CNN-BiLSTM achieved an SNR of 49.62 dB, RMSE of 0.002886, and PCC of 0.988945. The corresponding aggregate SNR values were 43.58 dB for the local PC-BiLSTMNet reimplementation, 43.85 dB for validation-selected Median filtering, and 45.12 dB for validation-selected VMD.

The physical-domain results in Table 2b evaluate the same reference signals and method outputs after restoring their magnetic-flux-density coordinates. WL-CNN-BiLSTM achieved an aggregate physical-domain SNR of 17.53 dB and RMSE of 1.48257 × 10^−10^ T. These physical-domain values are reported separately from the normalized-domain scores rather than interpreted as interchangeable measures.

**Figure 5:**
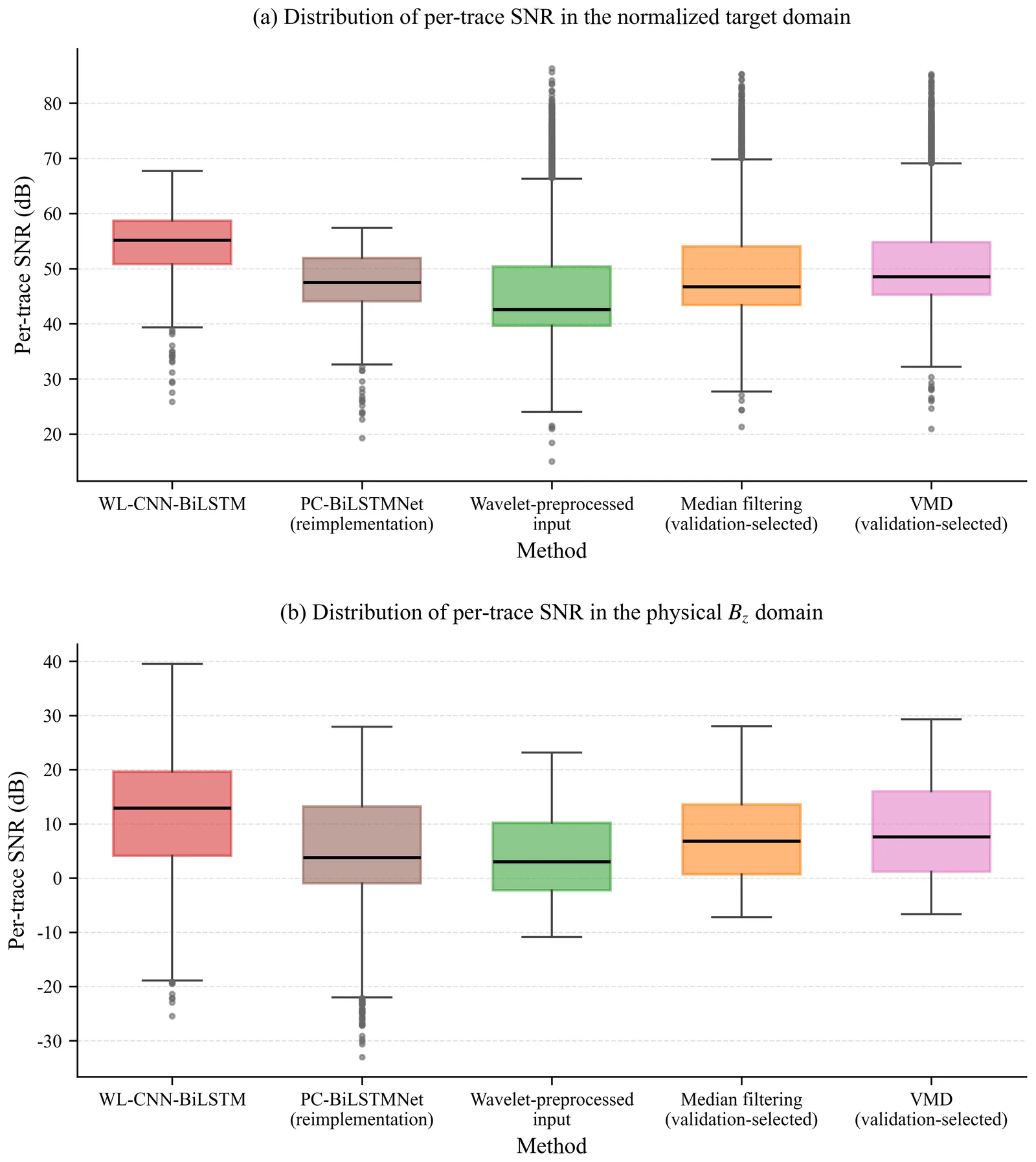
Distributions of per-trace SNR on the model-development test subset in (**a**) the common normalized clean-target domain and (**b**) the physical *B_z_* domain. Each box spans the 25th to 75th percentiles, denoted by Q1 and Q3, and the horizontal line indicates the median. The interquartile range is IQR = Q3 − Q1. Whiskers extend to the lowest and highest observed values within [Q1 − 1.5IQR, Q3 + 1.5IQR]; all observations outside these limits are plotted individually. Colors identify WL-CNN-BiLSTM (red), PC-BiLSTMNet (reimplementation; brown), Wavelet-preprocessed input (green), Median filtering (validation-selected; orange), and VMD (validation-selected; magenta). SNR is expressed in dB in both panels, and all 3000 traces are included for each configuration.

WL-CNN-BiLSTM obtained the highest aggregate SNR and PCC and the lowest aggregate RMSE among the evaluated configurations. Table 3 and Figure 5 characterize the accompanying variability across individual traces in both evaluation domains, using the same test subset, checkpoints, and conventional method settings as Table 2. All 3000 traces yielded finite SNR values for every configuration in both domains, and the boxplots retain all observations beyond the whiskers.

In the normalized clean-target domain, WL-CNN-BiLSTM had a mean per-trace SNR of 54.44 dB and a median of 55.17 dB, compared with its aggregate SNR of 49.62 dB in Table 2a. These quantities summarize different aspects of the same predictions and are not interchangeable. WL-CNN-BiLSTM had the highest mean and median among the evaluated configurations, but the validation-selected Median and VMD methods had higher P90 values. Its advantage therefore did not extend to every reported percentile.

In the physical *B_z_* domain, WL-CNN-BiLSTM also had the highest mean and median SNR, at 11.55 and 12.95 dB, respectively. However, its P10 was −3.41 dB and its minimum was −25.45 dB. The corresponding P10 and minimum were −1.25 and −7.22 dB for Median filtering and −0.99 and −6.65 dB for VMD. Thus, higher central values for WL-CNN-BiLSTM coexisted with less favorable lower-tail statistics than those of the validation-selected conventional methods. These distribution-level comparisons do not imply that the respective minima occurred on the same trace.

A negative physical-domain output SNR indicates that reconstruction-error energy exceeds clean-reference energy over the evaluated trace. It does not, by itself, establish deterioration relative to that trace’s input, which requires a paired comparison. Likewise, positive normalized-domain SNR values do not rule out large physical-domain relative errors, because the affine offset described in Section 3.1 changes the reference energy used in the SNR calculation.

To examine unfavorable reconstructions at the individual-trace level, we retrospectively selected two diagnostic cases from the completed WL-CNN-BiLSTM test predictions. Case A minimizes whole-trace SNR in the physical *B*_z_ domain, whereas Case B maximizes NRMSE over the 91 receiver times from 101 to 1001 μs. Ties were resolved by the lowest zero-based test-array index. The criteria selected two different traces, with indices 2222 and 1379, respectively. For each case, the clean reference, wavelet-preprocessed input, and all method outputs correspond to the same test trace. This diagnostic selection did not alter the trained models, the baseline configurations, or the reported test predictions.

**Figure 6:**
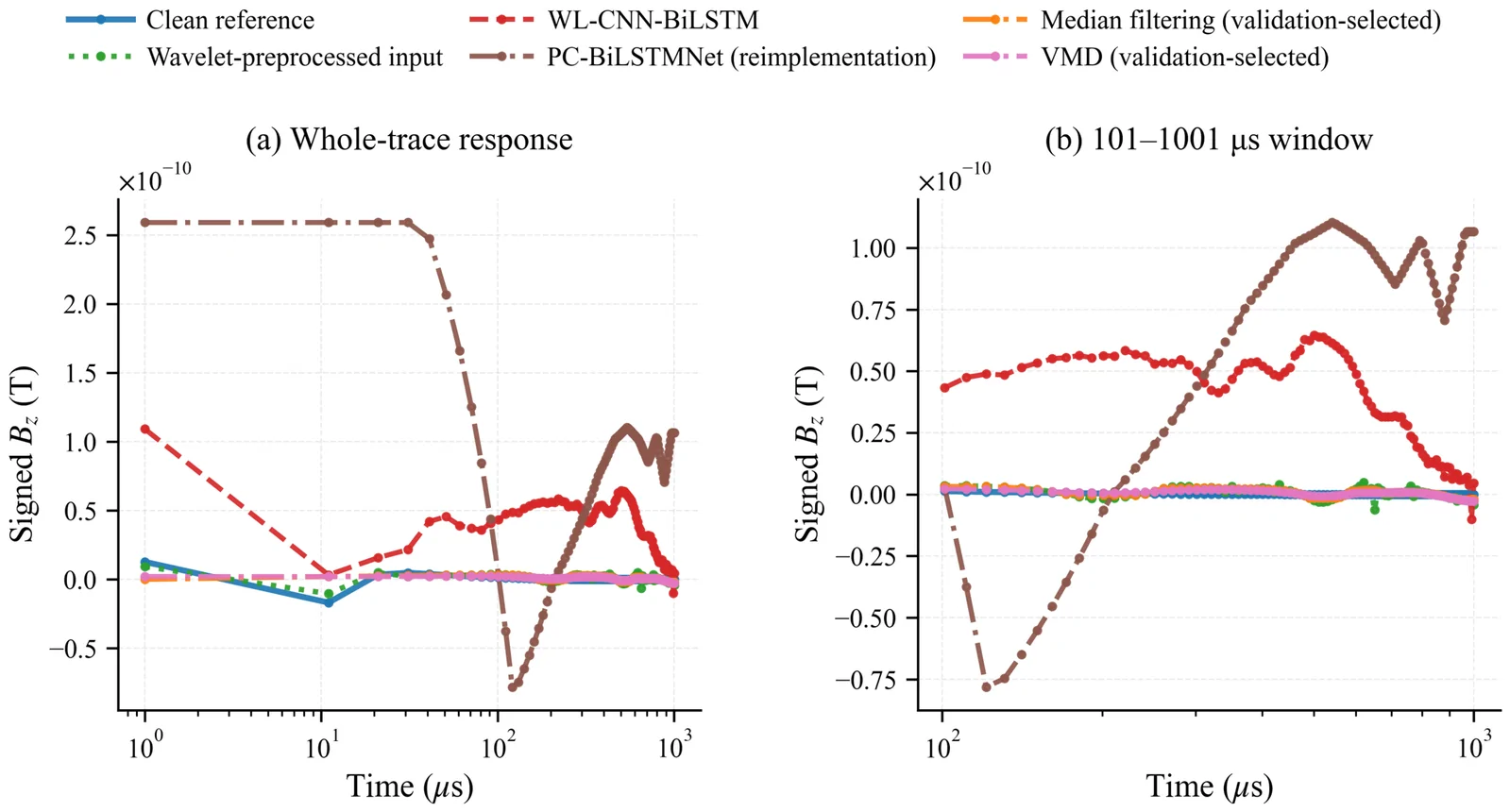
Failure case A, selected as the test trace with the lowest whole-trace physical-domain SNR for WL-CNN-BiLSTM (zero-based test-array index 2222). Panel (**a**) shows all 101 receiver times from 1 to 1001 μs, and panel (**b**) shows the 91 samples from 101 to 1001 μs. Curves represent the Clean reference (blue solid), Wavelet-preprocessed input (green dotted), WL-CNN-BiLSTM (red dashed), PC-BiLSTMNet (reimplementation; brown dash-dot), Median filtering (validation-selected; orange dash-dot), and VMD (validation-selected; magenta dash-dot). All curves correspond to the same trace. Time is displayed on a logarithmic axis, whereas signed *B_z_* amplitudes are displayed on a linear axis in T. Markers indicate the actual samples, and connecting lines do not introduce additional receiver times.

Case A, shown in Figure 6, had the lowest whole-trace physical-domain SNR for WL-CNN-BiLSTM, at −25.45 dB, compared with 0.63 dB for the wavelet-preprocessed input. On this same trace, the SNR values were −33.00 dB for PC-BiLSTMNet (reimplementation), −1.44 dB for Median filtering (validation-selected), and −0.57 dB for VMD (validation-selected). The paired comparison therefore demonstrates deterioration relative to the preprocessed input for WL-CNN-BiLSTM in this case. Its NRMSE over 101–1001 μs was 144.26. Approximately 90.02% of its total squared reconstruction error occurred in this evaluation window, whereas the first receiver sample contributed approximately 4.98%. The observed error was therefore not predominantly confined to the first sample; substantial amplitude discrepancies persisted over the 101–1001 μs window.

**Figure 7:**
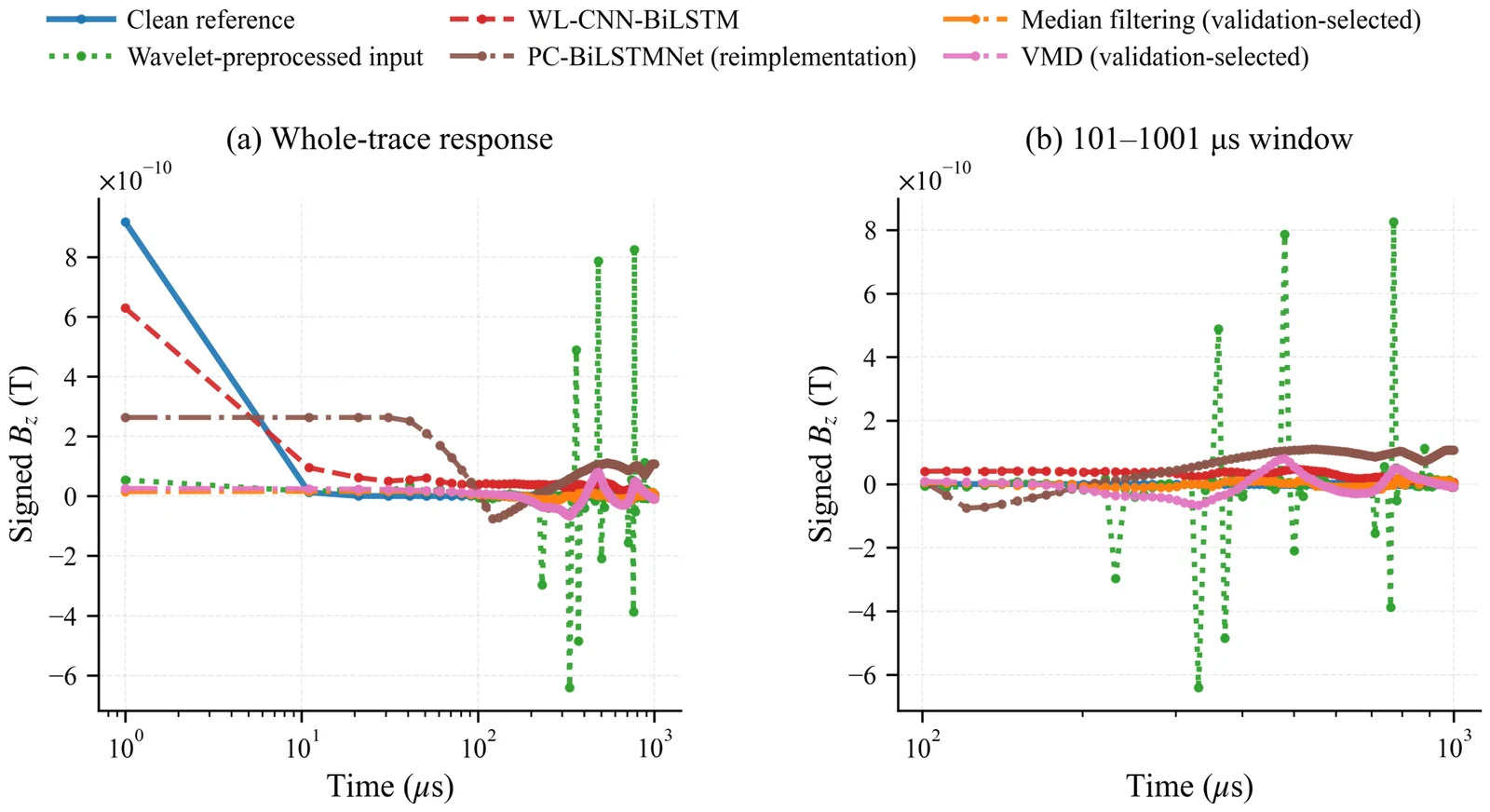
Failure case B, selected as the test trace with the largest physical-domain NRMSE over 101–1001 μs for WL-CNN-BiLSTM (zero-based test-array index 1379). Panel (**a**) shows all 101 receiver times from 1 to 1001 μs, and panel (**b**) shows the 91 samples from 101 to 1001 μs. The clean reference, wavelet-preprocessed input, and four method outputs correspond to the same trace, with the method names, colors, and line styles defined in Figure 6. Time is displayed on a logarithmic axis and signed *B_z_* on a linear axis in T. The near-zero reference in panel (**b**) should be interpreted together with the absolute-error and relative-error values reported in the text.

Case B, shown in Figure 7, had the largest NRMSE over 101–1001 μs for WL-CNN-BiLSTM. Its whole-trace SNR was 6.41 dB, compared with −5.91 dB for the wavelet-preprocessed input. Over the 101–1001 μs interval, the clean-reference RMS amplitude was 2.672 × 10^−16^ T and the WL-CNN-BiLSTM reconstruction RMSE was 3.003 × 10^−11^ T, yielding an NRMSE of 1.124 × 10^5^. This NRMSE was lower than the input value of 6.238 × 10^5^ but higher than the value of 3.415 × 10^4^ obtained by validation-selected Median filtering. The exceptionally small reference amplitude amplifies the relative-error measure, so its interpretation requires the absolute RMSE as well as the reference RMS. No system-specific detectability threshold has been established for this synthetic benchmark. These values therefore characterize reconstruction of the archived numerical target; the large NRMSE alone does not establish failure to recover a physically detectable signal within the evaluated 101–1001 μs window.

These retrospectively selected examples illustrate two distinct limitations: deterioration relative to the preprocessed input in Case A, and substantial relative error over 101–1001 μs despite improved whole-trace SNR in Case B. They are diagnostic examples rather than estimates of failure frequency across the test subset. Their interpretation is therefore considered together with the distributions in Table 3 and Figure 5. The observed discrepancies characterize the complete processing pipelines and do not, by themselves, isolate the causal contributions of preprocessing, normalization, and the neural-network components.

**Figure 8:**
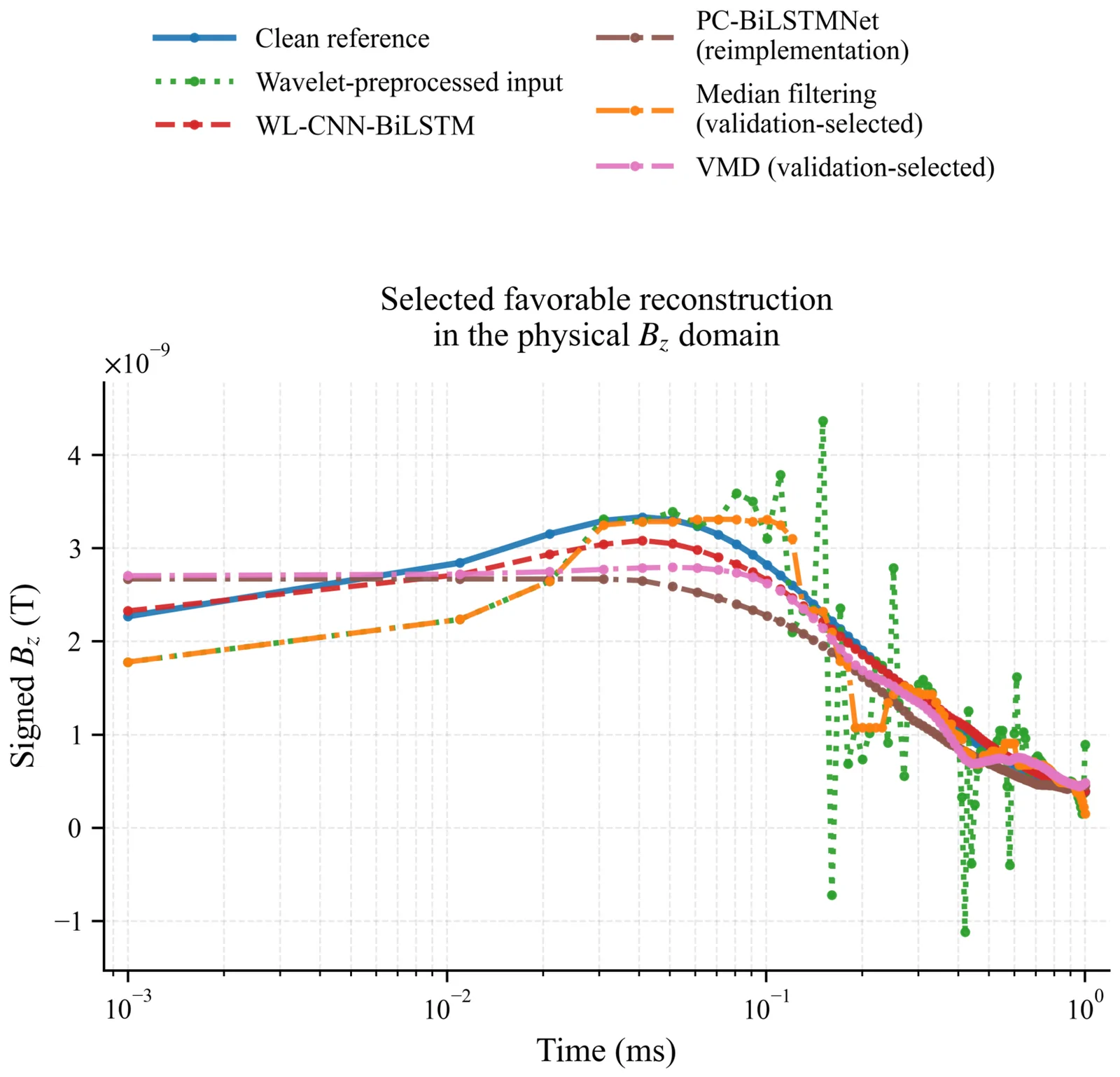
Selected favorable reconstruction example for WL-CNN-BiLSTM (zero-based test-array index 9), chosen using the saved per-trace physical-domain SNRs as described in the text. Curves show the clean reference, wavelet-preprocessed input, WL-CNN-BiLSTM, PC-BiLSTMNet (reimplementation), Median filtering (validation-selected), and VMD (validation-selected), using the same processing configurations and saved outputs as Table 2 and Table 3. The 101 receiver times span 1–1001 μs and are displayed in milliseconds on a logarithmic axis. The vertical axis shows signed *B*_z_ in T on a linear scale. Markers denote stored samples, and lines connect consecutive samples for visualization.

Figure 8 illustrates a selected favorable reconstruction by WL-CNN-BiLSTM from the stored benchmark test subset (zero-based test-array index 9). We selected the example retrospectively using the saved per-trace physical-domain SNRs: among traces with a WL-CNN-BiLSTM SNR of at least 20 dB and an advantage of at least 5 dB over each of the three comparison methods, we chose the lowest test-array index. For this trace, WL-CNN-BiLSTM achieved a whole-trace physical-domain SNR of 24.48 dB, compared with 15.00 dB for PC-BiLSTMNet (reimplementation), 15.54 dB for Median filtering (validation-selected), and 18.56 dB for VMD (validation-selected). The clean reference, wavelet-preprocessed input, and four method outputs correspond to the same trace and the processing configurations used in Table 2 and Table 3. All curves are displayed in physical *B*_z_ coordinates using the saved outputs and the matching inverse transformations described in Section 3.1.

**Figure 9:**
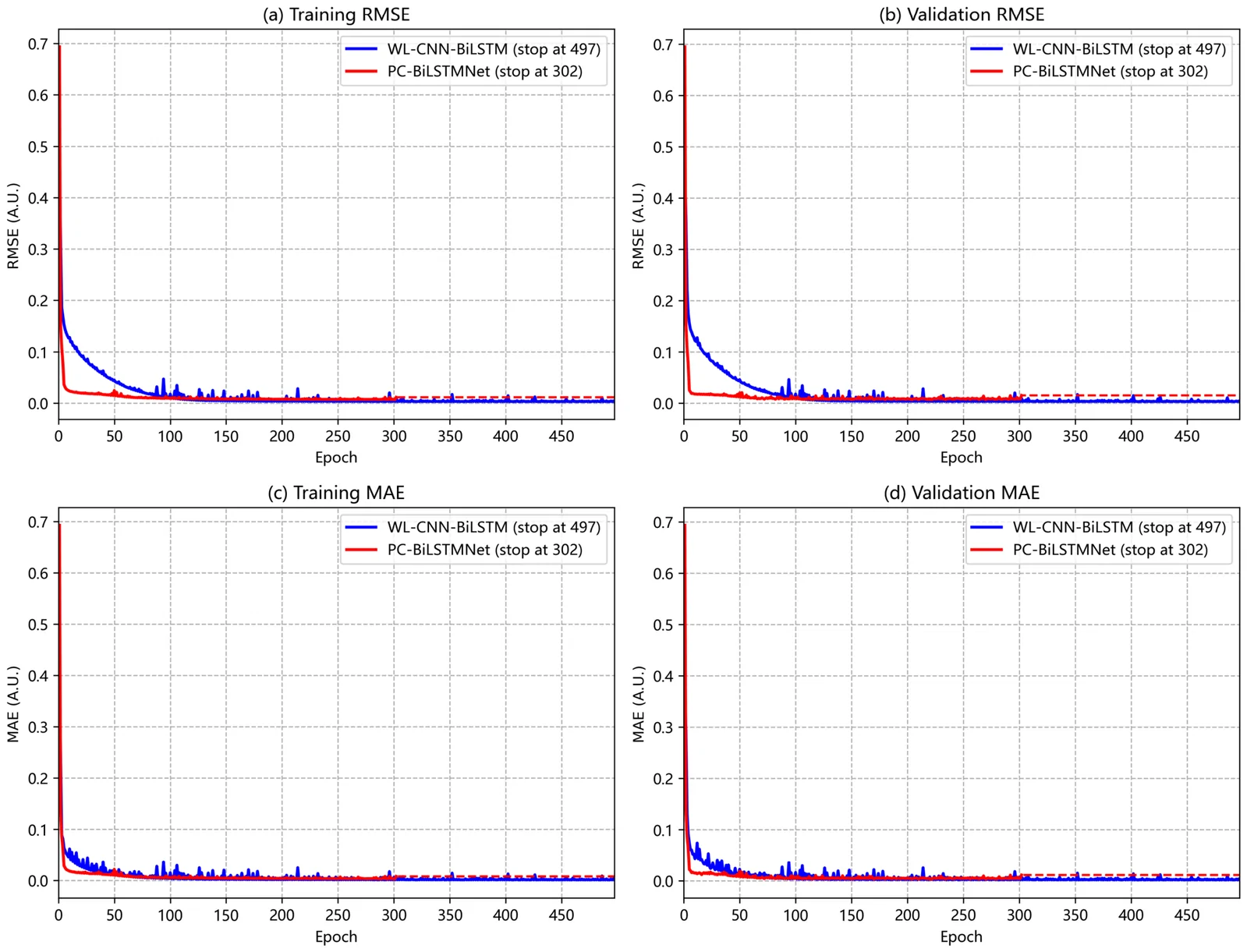
Training and validation reconstruction metrics for WL-CNN-BiLSTM and the local PC-BiLSTMNet reimplementation: (**a**) training RMSE, (**b**) validation RMSE, (**c**) training MAE, and (**d**) validation MAE. RMSE and MAE are evaluated in the normalized clean-target domain and expressed in arbitrary units (A.U.). Training stopped at epochs 497 and 302, respectively. Dashed extensions beyond a model’s stopping epoch repeat its final recorded value for visual comparison and do not represent additional training or validation evaluations.

Figure 9 shows the training and validation RMSE and MAE trajectories for the two implemented neural-network configurations. PC-BiLSTMNet shows a more rapid initial decrease in these metrics and stops at epoch 302, whereas WL-CNN-BiLSTM stops at epoch 497 and exhibits fluctuations during training. These epoch numbers indicate when training terminated, rather than the epochs of the validation-selected checkpoints used for test evaluation. Because the configurations used different optimizer settings and loss-term coefficients, the observed differences cannot be attributed solely to architectural complexity. The curves describe the recorded optimization trajectories; they do not establish the absence of overfitting or a causal explanation for the test-set differences in Table 2.

### 3.3. Ablation Study

We compared the full model with variants that disabled specified components or replaced the hybrid training objective with MSE. Each configuration was trained using three paired random-seed settings on the same stored training, validation, and test subsets, with the same batch size, optimizer settings, learning-rate schedules, maximum epoch limit, checkpoint-selection rule, and early-stopping patience, apart from the intended component or training-objective change.

In Table 4, “w/o internal wavelet layer” denotes removal of only the fixed, symmetric-extension wavelet-thresholding stage inside the network. The external periodization-based wavelet preprocessing and subsequent normalization were retained, and all ablation configurations received the same stored preprocessed inputs. The comparison therefore assesses the inclusion of the internal stage conditional on the external preprocessing; it does not assess removal of all wavelet processing or isolate the effect of the boundary-extension mode.

For every configuration, including the MSE-only variant, the training routine monitored the validation MSE defined in Section 2.5. It saved a configuration-specific checkpoint when that score improved and stopped according to the same validation-based rule. The saved checkpoint was reloaded before calculating the configuration’s test-set SNR. Test scores were used to report the completed runs and their differences relative to the full configuration; they were not supplied to the automatic stopping or checkpoint-selection rules. The configuration list was explicitly specified in the implementation and was not automatically modified in response to these test scores. The test results are summarized in Table 4. The “w/o temporal feature weighting” variant disables this feature multiplication while retaining the same optimizer and learning-rate settings; no separate learning-rate retuning was performed.

The full-model result in Table 2a and the repeated-seed results in Table 4 originate from separate experiments. Table 2a reports the model trained for the method-comparison benchmark, whereas Table 4 summarizes the full model and ablation variants evaluated with three paired random seeds. The repeated-seed statistics in Table 4 provide a descriptive estimate of variability within the archived ablation protocol.

Removing temporal feature weighting increased the mean physical-domain aggregate SNR by 0.6004 ± 0.0378 dB and the median per-trace SNR by 0.6798 ± 0.5009 dB, while reducing the median NRMSE over 101–1001 μs by 0.0904 ± 0.0333 relative to the full model. These descriptive paired differences across three seeds indicate that the implemented weighting did not improve the evaluated reconstruction measures.

Table 5 reports the exponent sensitivity experiment on the 8000-training/1500-validation layered-earth dataset. The matched *p* = 0 and *p* = 1.5 comparison showed that *p* = 0 achieved a validation median physical-domain SNR of 8.6074 dB and a median NRMSE over 101–1001 μs of 6.0221, compared with 6.9161 dB and 7.6573 for *p* = 1.5. The previously completed *p* = 1.0 run yielded a lower interval NRMSE of 3.8273, whereas *p* = 2.0 performed worse on both reported measures. These results do not support a late-time fidelity benefit from the positive power-law weighting and do not show a monotonic relationship between *p* and reconstruction quality. The *p* = 1.5 configuration remained the prespecified frozen reference for the subsequent sensitivity and stress tests rather than an exponent selected as optimal by this comparison.

### 3.4. Field-Data Transfer Example

The field measurements were acquired using the iFTEM-II fixed-wing time-domain airborne electromagnetic system with a half-sinusoidal transmitter waveform. The models trained solely on synthetic data were applied without fine-tuning to the selected field-data windows to examine their qualitative transfer behavior. The underlying field dataset and further acquisition details are not publicly disclosed because of confidentiality restrictions. The measured quantity was vertical magnetic flux density *B*_z_, supplied in nT and converted to T using 1 nT = 10^−9^ T before wavelet preprocessing and normalization. No integration or differentiation was applied to the measured signal.

The curve labeled “engineering proxy” was supplied with the measurements and is retained as a qualitative comparison curve. Its generation procedure and independent amplitude calibration have not been established. The existing display procedure applies the same numerical scale factor of 10^−9^ to this curve. Agreement with it is therefore interpreted as similarity to the supplied comparison curve, rather than recovery of an independently verified noise-free field response.

**Figure 10:**
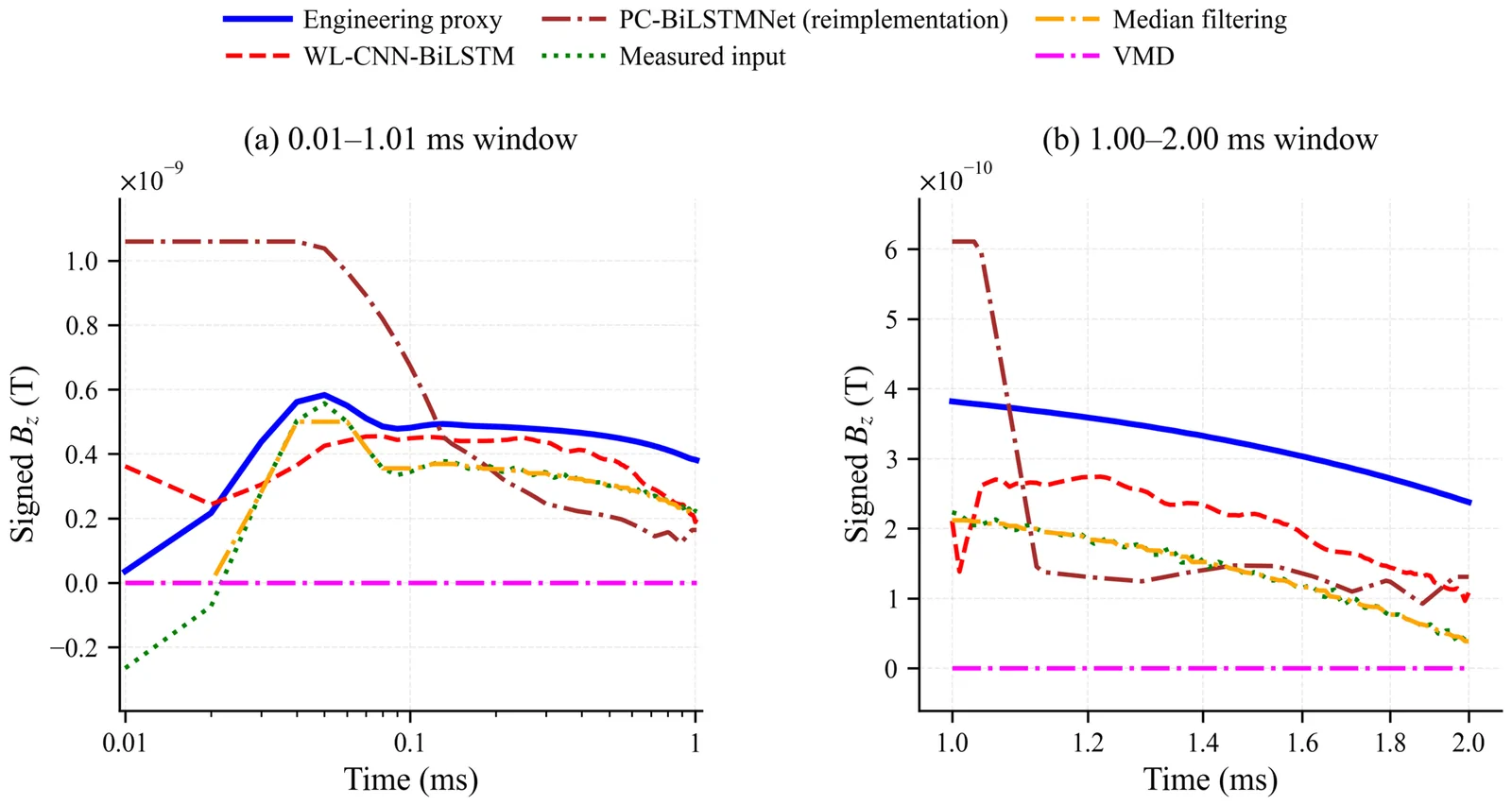
Qualitative comparison of measured and processed field signals in two separately processed 101-sample windows: (**a**) 0.01–1.01 ms and (**b**) 1.00–2.00 ms. Time is displayed on a logarithmic axis and refers to the supplied record coordinate, whose relationship to transmitter shutoff is unconfirmed. Signed measured and reconstructed (*B*_z_) amplitudes are displayed on a linear axis in T, retaining zero and negative values. The saved VMD output is zero throughout both windows and is shown as a magenta dash-dot line at zero. The engineering-proxy curve follows the numerical display scaling described in Section 3.4 and is not an independently verified noise-free reference.

Figure 10 presents two separately processed 101-sample windows: 0.01–1.01 ms and 1.00–2.00 ms. These intervals refer to the time coordinate supplied with the measurements. The relationship between its time origin and transmitter current shutoff has not been established, so these intervals cannot be directly identified with the post-shutoff receiver times used for synthetic training. Accordingly, the second window cannot be classified as a verified extrapolation beyond the model’s physical-time training support.

WL-CNN-BiLSTM produces a smoother displayed curve than the measured input in Figure 10a and lies closer to the supplied engineering proxy than PC-BiLSTMNet and Median filtering over much of Figure 10b. In panel (a), PC-BiLSTMNet has higher displayed amplitudes than the proxy near the beginning of the window, while Median filtering shows a local deviation near 0.03 ms. The zero-valued VMD output is specific to the saved field-processing results and should not be interpreted as representative of VMD performance in general. Differences between WL-CNN-BiLSTM and the proxy remain in both panels. These observations characterize the displayed waveforms under the stated scaling; the undocumented construction and calibration of the proxy prevent their interpretation as verified differences in true field reconstruction accuracy.

### 3.5. Sensitivity to Impulsive Contamination

The synthetic impulse-probability range of 10–30% was used to define a severe, controlled corruption setting rather than an empirically calibrated representation of typical field contamination.

We examined impulse-probability sensitivity using a separately trained WL-CNN-BiLSTM configuration based on a numerically checked layered-earth forward model. This configuration used 8000 synthetic training traces, 1500 validation traces, and a temporal feature-weighting exponent of 1.5. Its input and target scalers were fitted to the training subset. The weights and scalers were frozen before the sensitivity analysis, with no further training, fine-tuning, or checkpoint selection. This configuration is distinct from the model used for the 20,000-sample development benchmark.

The sensitivity analysis used 500 newly generated clean traces at impulse probabilities of 0%, 5%, 10%, 20%, 30%, and 40%. The clean response and the Gaussian and sferic realizations for each trace were held fixed across conditions. Impulse realizations were generated using predefined condition-specific random seeds and the signed-uniform, clean-peak-scaled amplitude definition described in Section 2.2. The impulse masks were not nested across probabilities, and the amplitude factors were not held fixed across conditions. This experiment therefore compares stochastic corruption settings rather than a deterministic progression obtained by adding impulses to an otherwise unchanged impulse realization. At 0% impulse probability, the Gaussian and sferic components remained present.

Table 6 summarizes per-trace performance in the physical *B_z_* domain: median, P10, and P90 whole-trace SNR, median whole-trace RMSE, and median NRMSE over 101–1001 μs, using the metric definitions in Section 3.1.

The median output SNR varied non-monotonically with impulse probability, reaching 8.27 dB at 5% and decreasing to 4.80 dB at 40%. The output SNR P10 remained approximately −44.95 dB across all conditions, whereas the raw-input SNR P10 changed from −6.08 dB at 0% to −14.10 dB at 40%. The higher median output SNR therefore coexisted with a substantially poorer lower tail and did not indicate consistent improvement across traces. The median NRMSE over 101–1001 μs ranged from 9.64 to 10.65, indicating substantial relative reconstruction errors within this evaluation window.

Analysis of the saved paired outputs showed that the same 50 traces occupied the lowest 10% of whole-trace output SNR values in all six conditions. These were also the 50 traces with the lowest clean-signal energy. Within this subset, the median fraction of total squared reconstruction error contributed by the first receiver sample was approximately 97.71%, compared with approximately 2.21% from the 101–1001 μs evaluation window. The nearly invariant lower tail therefore reflects a systematic reconstruction failure affecting weak signals, rather than reliable denoising across impulse conditions.

We performed an additional evaluation-interval sensitivity analysis using the saved clean references and predictions, without retraining, new inference, or filtering. The full 101-sample metrics were first reproduced, and the metrics were then recalculated over the remaining 100 samples from 11 to 1001 μs. All 500 traces were retained in every condition. After excluding the first sample, median SNR ranged from −7.3768 to −5.8800 dB, and P10 ranged from −40.0392 to −40.0148 dB. Thus, severe lower-tail errors persisted. The lowest 50 traces under the two evaluation intervals overlapped in only 29 cases, so the change in P10 does not represent a paired improvement for an unchanged tail subset. The NRMSE values over 101–1001 μs were unchanged because this evaluation window was unaffected. Table S1a reports both evaluation intervals; Table 6 retains the full 101-sample metrics.

Excluding the first sample removes both reference-signal energy and reconstruction-error energy. Across the 500 clean traces, the median fraction of reference energy at the first sample was 95.44%, so a reduction in error energy alone does not imply a higher SNR. The first sample occurs at 1 μs after the ideal step-off, but its timing does not establish that the waveform idealization caused the amplitude mismatch. The saved inputs and outputs also do not identify the causal contributions of scaler range, preprocessing, or the neural network. No controlled waveform or scaler intervention was performed in this diagnostic analysis.

### 3.6. Out-of-Distribution Evaluation and Noise-Curriculum Robustness

The structural OOD and noise-stress tests reported in Table 7 used the separately trained and frozen WL-CNN-BiLSTM configuration described in Section 2.2 and Section 3.5. They did not use the model-development checkpoint evaluated in Table 2 and Table 3. No further fitting or checkpoint selection was performed for these tests.

A separate, previously completed transfer evaluation applied the archived model-development checkpoint and its original scalers to 500 independent traces generated with the layered-earth procedure described in Section 2.2. Without retraining or fine-tuning, the median per-trace physical-domain SNR was −7.10 dB, compared with −7.84 dB for the raw noisy input. However, the median NRMSE over 101–1001 μs was 104.56, compared with 36.71 for the raw input. Thus, the modest improvement in whole-trace median SNR did not establish reliable reconstruction over the 101–1001 μs evaluation window under this transfer. This evaluation used a different 500-trace set from the paired conditions in Table 6 and Table 7b.

For the structural tests, we evaluated explicit one-dimensional changes because the procedural labels in Section 2.2 do not define independent three-dimensional geological classes.

The training support comprised 3–8 layers, finite-layer thicknesses of 20–500 m, and conductivities of 10^−5^–1 S/m. Each structural OOD family contained 200 new models with the training noise distribution retained. G1 introduced an interior conductive layer of 5–<20 m and 0.1–1 S/m within a 10^−5^–10^−2^ S/m background. G2 introduced an interior resistive layer of the same thickness range and 10^−5^–10^−4^ S/m within a 10^−2^–1 S/m background. G3 used 9–12 layers with the training thickness and conductivity ranges. G1 and G2 jointly test thin-layer and background-contrast configurations, not thickness alone. An independent 1000-trace in-distribution (ID) blind set provided the structural-test reference.

To evaluate robustness to mismatch between the training noise curriculum and test-time contamination, we performed controlled noise-stress experiments using 500 paired clean traces. The paired reference condition used a Gaussian standard deviation equal to 30% of the pointwise absolute clean amplitude, an impulse probability of 20%, and an impulse peak scale of 100%.

We examined three forms of curriculum mismatch. First, we tested noise amplitudes or occurrence rates beyond the training ranges. These included Gaussian scales of 60%, 75%, and 100%; impulse peak scales of 200%, 250%, and 300%; and impulse probabilities of 40% and 50%. Second, we tested noise types absent from training by adding random-phase sinusoids at 50 Hz, 1 kHz, and 5 kHz. Their amplitudes were equal to 20% of the clean-signal peak. Third, we tested an atypical combined condition with a 75% Gaussian scale and a 200% impulse peak scale.

Unless explicitly changed, the remaining noise components and underlying random draws were retained across paired conditions. All evaluations were performed in the physical *B_z_* domain using the metrics defined in Section 3.1.

The stress tests show that degradation depends strongly on the type of mismatch. Increasing the Gaussian scale beyond the 50% training maximum produced relatively moderate changes in median SNR. The median was 7.26 dB in the paired reference condition and decreased to 6.59, 6.49, and 6.50 dB at Gaussian scales of 60%, 75%, and 100%, respectively. Increasing impulse peak amplitudes beyond the training range caused substantially greater degradation. The median SNR decreased to 1.61 dB at an impulse peak scale of 200%, −0.91 dB at 250%, and −2.66 dB at 300%. Increasing impulse probability to 40% and 50% reduced the median SNR to 5.20 and 4.70 dB, respectively.

For noise types absent from training, the added 50 Hz, 1 kHz, and 5 kHz sinusoids yielded median SNR values of −1.56, 1.48, and 2.69 dB, respectively. The combined condition with a 75% Gaussian scale and a 200% impulse peak scale yielded a median SNR of 1.73 dB. The 50 Hz result should be interpreted cautiously. The 1 ms observation span contains only 0.05 of a 50 Hz cycle, so the added sinusoid represents a local trend within this window rather than a complete power-line waveform.

The structural OOD results in Table 7a also show strongly configuration-dependent performance. G1, which combines a thin conductive layer with a resistive background, produced the largest degradation among the structural families: median SNR was −21.01 dB, compared with 5.91 dB for the independent ID reference, and median NRMSE over 101–1001 μs was 47.539, compared with 7.402. This is a substantial failure of physical-amplitude reconstruction under the tested conductive-layer configuration. G2 yielded a median SNR of 12.76 dB and a median NRMSE over 101–1001 μs of 0.469, whereas G3 yielded 4.16 dB and 13.766, respectively. G1 and G2 jointly change thin-layer and background-contrast configurations, so their differences cannot be attributed to layer thickness alone. Comparisons with ID are differences between independent family summaries, not paired effects or evidence of general robustness to thin layers.

We also applied the first-sample exclusion diagnostic to all 13 noise conditions in Table 7b, using the saved predictions and retaining all 500 traces per condition. After exclusion, the reference median SNR was −6.5637 dB. The corresponding values at Gaussian scales of 60%, 75%, and 100% were −6.4797, −6.4491, and −6.4022 dB. These changes remained small, but their direction relative to the reference differed from the full-interval results. By contrast, increasing the impulse peak scale to 200%, 250%, and 300% reduced median SNR to −9.4228, −11.4393, and −12.9067 dB, respectively. The degradation associated with increasing impulse amplitude therefore persisted after removal of the first sample.

Across the 13 conditions, P10 remained between −40.0456 and −40.0009 dB after exclusion. The severe lower-tail failure was therefore not confined to the first receiver sample, although that sample dominated the squared error of the original weakest-trace subset. The NRMSE values over 101–1001 μs remained unchanged. Table 7b retains the full 101-sample evaluation, and Table S1b reports the interval comparison separately.

These results show that some whole-trace summaries and condition rankings depend on the evaluation interval. Nevertheless, the conductive-layer failure, the degradation under increasing impulse amplitude, and the substantially lower-tail errors and relative errors over 101–1001 μs preclude a claim of uniform robustness outside the training distribution. The diagnostic does not establish the physical or computational cause of the first-sample mismatch.

### 3.7. Independent Layered-Earth Blind-Test Comparison

We additionally report a previously completed comparison on an independent 1000-trace layered-earth blind-test set. WL-CNN-BiLSTM used the separately trained 8000-training/1500-validation configuration described in Section 2.2, with *p* = 1.5 and scalers fitted only to its training subset. Its checkpoint and scalers were frozen before this comparison. The WL-CNN-BiLSTM results on this blind set also provide the ID reference in Table 7a; they are not an additional independent replication of that reference.

Conventional method settings were selected exclusively on a separate 1000-trace baseline-validation set by maximizing median per-trace physical-domain SNR. The primary raw-input comparison selected a seven-point Median kernel and VMD with *K* = 2, *α* = 5000, *τ* = 0, and reconstruction after removal of the last returned mode. The separately selected wavelet-preprocessed variants used an 11-point Median kernel and the same VMD settings. Median filtering used zero padding. VMD used per-trace peak scaling, a stopping tolerance of 10^−7^, and extension of the 101-point input to 102 points followed by trimming after reconstruction. All comparison settings were frozen before generating the blind-test set.

Each processing pipeline started from the same raw observation. WL-CNN-BiLSTM retained its prescribed wavelet preprocessing and normalization, whereas the primary Median and VMD methods operated directly on raw observations. Wavelet-preprocessed conventional results are reported separately in Table 8b. The archived PC-BiLSTMNet checkpoint was also evaluated as a transfer reference; it was not retrained on the layered-earth distribution and therefore does not provide a comparison under matched training conditions.

WL-CNN-BiLSTM achieved a higher median whole-trace SNR than the validation-selected raw-input Median and VMD pipelines. Its substantially poorer lower tail, however, was accompanied by a lower arithmetic mean SNR than those of both conventional methods. Median filtering also achieved a much lower median NRMSE over 101–1001 μs. The results therefore support a median whole-trace reconstruction advantage under this protocol, while demonstrating that the advantage does not extend to all distributional summaries or to interval-restricted relative error.

## 4. Discussion

WL-CNN-BiLSTM achieved the strongest aggregate reconstruction metrics among the tested archived-benchmark configurations. The independent layered-earth comparison additionally showed a median whole-trace SNR advantage over validation-selected Median filtering and VMD, accompanied by poorer lower-tail performance. This contrast highlights the value of evaluating reconstruction across traces and receiver-time intervals alongside aggregate scores. The archived results concern numerical targets affected by the forward-model artifacts documented in Section 2.2.

To address whole-corpus scaler fitting, we retrained the archived-benchmark model with scalers fitted only to its 14,000 training traces. The original split, architecture, batch size, loss, and stopping settings were retained. Training reached 800 epochs, and the validation-selected checkpoint achieved aggregate SNRs of 50.0126 dB in the normalized domain and 17.9291 dB in the physical domain, with physical RMSE of 1.41626 × 10^−10^ T. These modest improvements over Table 2 reflect both the revised preprocessing and a new random seed, rather than an isolated scaler effect.

The matched *p* = 0 comparison and three-seed ablation did not establish a late-time fidelity benefit from temporal feature weighting. The layered-earth stress tests identified sensitivity to conductive-layer configurations and strong impulsive contamination. These tests characterize the separately trained configuration; the full stress suite was not repeated for the archived model or its scaler retraining.

The field example illustrates transfer behavior through qualitative waveform comparisons with an engineering proxy of unverified provenance and calibration. Further validation should incorporate characterized receiver timing, finite transmitter turn-off, and responses involving induced polarization, superparamagnetism, or magnetic-permeability contrasts. These extensions would better connect the evaluated conductivity-dependent simulations to field acquisition conditions.

## 5. Conclusions

This study developed a WL-CNN-BiLSTM framework for AEM/TEM denoising by combining fixed wavelet preprocessing, multi-scale convolution, residual and skip connections, bidirectional LSTM modules, and a hybrid reconstruction objective. A positional temporal feature-weighting module was retained as part of the evaluated implementation, but the matched *p* = 0 comparison and three-seed ablation did not establish a fidelity benefit attributable to this weighting.

Although per-trace analysis revealed uneven reconstruction quality, including large relative errors over 101–1001 μs for some traces, WL-CNN-BiLSTM achieved an aggregate normalized-domain SNR of 49.62 dB, RMSE of 0.002886, and PCC of 0.988945 on the 3000-trace model-development test subset. These results characterize the aggregate reconstruction performance of the evaluated benchmark configuration.

Transfer evaluations of the archived model and stress tests of the separate layered-earth configuration identified distribution-dependent failure modes. Complete retraining with training-only scalers yielded slightly higher aggregate SNRs under a new random seed, as discussed in Section 4. Future work should assess additional survey systems, transmitter waveforms, physical response mechanisms, and the effects of denoising on downstream inversion.

## Figures and Tables

**Table 1:** Baseline configurations used for the 20,000-sample model-development benchmark. Median filtering and VMD settings were selected on the validation subset.

Method	Description and Configuration
Median Filtering	Validation-selected kernel size: 11 samples; zero-padding boundary treatment.
VMD	Validation-selected *K* = 2, *α* = 2000, and *τ* = 0; stopping tolerance 10^−7^ on the peak-scaled input; reconstruction by summing the modes remaining after removal of the last returned mode.
PC-BiLSTMNet (reimplementation)	Local PyTorch reimplementation based on Cheng and Wu [18]; three parallel convolutional branches with kernel sizes 3/5/7; eight-head attention at branch and fused-feature levels; two-layer BiLSTM with 32 hidden units per direction; linear output projection and interpolation to 101 samples.

**Note:** The listed traditional-method settings apply to the model-development benchmark and are not universal configurations for all experiments. Validation-only selection of these parameters does not remove the benchmark’s forward-modeling limitations or the use of whole-corpus statistics in its retained scalers.

**Table 2:** Aggregate reconstruction metrics on the model-development test subset: (**a**) the common normalized clean-target domain and (**b**) the corresponding physical *B_z_* domain. Each metric is calculated over all 3000 traces and 101 receiver-time samples per trace. The two panels evaluate the same reference signals and method outputs.

(**a**) Common normalized clean-target domain			
**Method**	**Aggregate SNR (dB)**	**Aggregate RMSE (dimensionless)**	**Aggregate PCC**
WL-CNN-BiLSTM	49.62	0.002886	0.988945
PC-BiLSTMNet (reimplementation)	43.58	0.005783	0.949431
Wavelet-preprocessed input	39.87	0.008864	0.897207
Median filtering (validation-selected)	43.85	0.005607	0.953267
VMD (validation-selected)	45.12	0.004841	0.964726
(**b**) Physical *B_z_* domain			
**Method**	**Aggregate SNR (dB)**	**Aggregate RMSE (T)**	**Aggregate PCC**
WL-CNN-BiLSTM	17.53	1.48257 × 10^−10^	0.988945
PC-BiLSTMNet (reimplementation)	11.49	2.97107 × 10^−10^	0.949431
Wavelet-preprocessed input	7.78	4.55356 × 10^−10^	0.897207
Median filtering (validation-selected)	11.76	2.88034 × 10^−10^	0.953267
VMD (validation-selected)	13.04	2.48709 × 10^−10^	0.964726

**Note:** “Wavelet-preprocessed input” denotes the stored input after external wavelet preprocessing, without subsequent neural-network, Median, or VMD processing; it is not the raw noisy observation. Median and VMD use the validation-selected configurations in Table 1. Normalized-domain RMSE is dimensionless, whereas physical-domain RMSE is expressed in T. Aggregate metrics are not averages of per-trace metrics.

**Table 3:** Distribution of per-trace SNR on the 3000-trace model-development test subset: (**a**) the common normalized clean-target domain and (**b**) the physical *B_z_* domain. All numerical entries are expressed in dB.

(**a**) Common normalized clean-target domain						
**Method**	**Mean**	**SD**	**Median**	**P10**	**P** **9** **0**	**Minimum**
WL-CNN-BiLSTM	54.44	5.27	55.17	47.24	60.60	25.84
PC-BiLSTMNet (reimplementation)	47.52	4.51	47.51	42.10	52.84	19.26
Wavelet-preprocessed input	46.70	10.73	42.59	37.51	65.41	15.05
Median filtering (validation-selected)	50.33	10.29	46.76	41.03	67.93	21.33
VMD (validation-selected)	51.64	9.69	48.54	42.94	68.33	20.95
(**b**) Physical *B_z_* domain						
**Method**	**Mean**	**SD**	**Median**	**P10**	**P** **9** **0**	**Minimum**
WL-CNN-BiLSTM	11.55	10.91	12.95	−3.41	24.50	−25.45
PC-BiLSTMNet (reimplementation)	4.63	11.15	3.81	−10.40	19.55	−33.00
Wavelet-preprocessed input	3.81	7.11	3.04	−5.17	13.46	−10.87
Median filtering (validation-selected)	7.44	7.18	6.82	−1.25	17.18	−7.22
VMD (validation-selected)	8.74	8.13	7.59	−0.99	20.18	−6.65

**Note:** SD denotes the sample standard deviation, calculated with a denominator of *M* − 1, where *M* = 3000 is the number of traces. P10 and P90 are the 10th and 90th percentiles, calculated by linear interpolation of the sorted per-trace SNR values. The mean is the arithmetic mean of the individual SNR values in dB, not the aggregate SNR in Table 2. Each configuration’s minimum is determined separately and need not correspond to the same test trace. “Wavelet-preprocessed input” has the same meaning as in Table 2.

**Table 4:** Three-seed repeated ablation results under the archived benchmark data and preprocessing configuration. Values are reported as mean ± sample standard deviation across three paired random seeds. SNR values are reported in both normalized clean-target and physical *B*_z_ domains.

Configuration	Normalized Aggregate SNR (dB)	Physical Aggregate SNR (dB)	Median per-Trace SNR (dB)	Median NRMSE over 101–1001 μs
full model	48.9173 ± 0.1263	16.8337 ± 0.1263	12.0978 ± 0.2855	0.4616 ± 0.0689
w/o internal wavelet layer	50.4966 ± 0.1549	18.4131 ± 0.1549	14.2826 ± 0.7088	0.3372 ± 0.0546
w/o skip connections	49.6270 ± 0.3955	17.5434 ± 0.3955	13.0852 ± 0.4268	0.4071 ± 0.0395
w/o temporal feature weighting	49.5177 ± 0.1625	17.4341 ± 0.1625	12.7775 ± 0.2344	0.3712 ± 0.0373
w/o multi-scale fusion	50.1790 ± 0.5442	18.0954 ± 0.5442	14.0691 ± 0.9651	0.3297 ± 0.0952
w/o hybrid loss (MSE only)	48.8114 ± 0.2938	16.7278 ± 0.2938	11.4716 ± 1.3413	0.5475 ± 0.1472

**Table 5:** Validation sensitivity to the temporal feature-weighting exponent *p* for the separate layered-earth configuration. SNR is evaluated per trace in the physical *B_z_* domain. The interval-restricted NRMSE and amplitude-ratio statistics use the same 101–1001 μs evaluation window.

Exponent *p*	Training Traces	Validation Traces	Validation Median SNR (dB)	Validation Median NRMSE	Validation Median NRMSE over 101–1001 μs	Median Amplitude Ratio over 101–1001 μs
0	8000	1500	8.6074	0.3712	6.0221	6.9149
1.0	8000	1500	8.3331	0.3831	3.8273	3.9034
1.5	8000	1500	6.9161	0.4510	7.6573	8.3081
2.0	8000	1500	−2.4821	1.3308	9.6835	10.3202

**Note:** The *p* = 0 and *p* = 1.5 entries form a matched single-seed comparison. The *p* = 1.0 and *p* = 2.0 entries are single-run sensitivity results. The *p* = 0 comparison does not provide a cross-seed uncertainty estimate.

**Table 6:** Impulse-probability sensitivity of the separately trained and frozen WL-CNN-BiLSTM configuration. Statistics are calculated across the same clean traces under each noise condition in the physical *B_z_* domain.

Impulse Probability (%)	Median SNR (dB)	SNR P10 (dB)	SNR P90 (dB)	Median RMSE (T)	Median NRMSE over 101–1001 μs
0	6.04	−44.95	16.74	2.294 × 10^−11^	9.886
5	8.27	−44.95	19.44	2.212 × 10^−11^	9.915
10	7.77	−44.95	17.84	2.239 × 10^−11^	9.737
20	5.81	−44.95	18.51	2.256 × 10^−11^	10.574
30	6.90	−44.95	16.61	2.256 × 10^−11^	9.643
40	4.80	−44.95	15.60	2.302 × 10^−11^	10.651

**Note:** P10 and P90 are the 10th and 90th percentiles of per-trace SNR, calculated by linear interpolation of the sorted values. All 500 traces yielded finite SNR values in each condition. The P10 values differ by less than 0.0004 dB across conditions and therefore all round to −44.95 dB at the displayed precision.

**Table 7:** OOD evaluation of the separately trained, frozen WL-CNN-BiLSTM configuration: (**a**) structural shifts relative to an independent ID set; (**b**) paired noise-reference and stress conditions. SNR statistics are in dB; NRMSE values are dimensionless ratios.

(**a**) Structural OOD evaluation				
**Test family**	**Median SNR (dB)**	**Δ median SNR vs. ID (dB)**	**Median NRMSE**	**Median NRMSE over 101–1001 μs**
ID reference	5.91	0.00	0.506	7.402
Thin conductive layer (G1)	−21.01	−26.92	11.237	47.539
Thin resistive layer (G2)	12.76	+6.85	0.230	0.469
9–12 layers (G3)	4.16	−1.75	0.623	13.766
(**b**) Noise stress evaluation				
**Noise condition**	**Median SNR (dB)**	**SNR P10 (dB)**	**SNR P90 (dB)**	**Median NRMSE over 101–1001 μs**
Paired reference	7.26	−44.95	17.35	9.890
Gaussian scale 60%	6.59	−44.95	17.33	10.009
Gaussian scale 75%	6.49	−44.95	16.64	9.893
Gaussian scale 100%	6.50	−44.95	16.43	9.780
Impulse peak scale 200%	1.61	−44.95	13.44	11.447
Impulse peak scale 250%	−0.91	−44.95	12.14	12.743
Impulse peak scale 300%	−2.66	−44.95	11.25	14.109
Impulse probability 40%	5.20	−44.95	16.52	10.044
Impulse probability 50%	4.70	−44.95	16.32	10.813
Added 50 Hz sinusoid	−1.56	−44.95	13.56	15.004
Added 1 kHz sinusoid	1.48	−44.95	13.66	11.496
Added 5 kHz sinusoid	2.69	−44.95	14.52	9.767
Gaussian 75% + impulse peak scale 200%	1.73	−44.95	13.61	11.470

**Note:** In (a), Δ denotes the difference between independently calculated family and ID medians, not a paired effect. In (b), unspecified settings retain their reference values. Impulse peak scales specify the bounds of signed-uniform amplitudes relative to the clean peak, not an identical amplitude for every spike. P10 and P90 denote the 10th and 90th percentiles.

**Table 8:** Previously completed independent layered-earth blind-test comparison: (**a**) raw-observation processing pipelines and an archived-checkpoint transfer reference; (**b**) supplementary wavelet-preprocessed conventional pipelines. All statistics are calculated across the same 1000 traces in physical *B*_z_ coordinates.

(**a**) Raw-observation processing pipelines					
**Method**	**Mean SNR (dB)**	**Median SNR (dB)**	**SNR P10 (dB)**	**Median RMSE (T)**	**Median NRMSE over 101–1001 μs**
Raw input	−7.33	−7.82	−11.84	6.194 × 10^−11^	34.378
WL-CNN-BiLSTM	−6.37	5.91	−43.93	2.296 × 10^−11^	7.402
Median filtering (validation-selected)	2.08	0.39	−0.18	2.423 × 10^−11^	0.213
VMD (validation-selected)	1.22	−0.05	−2.54	2.704 × 10^−11^	8.883
PC-BiLSTMNet (reimplementation; archived-checkpoint transfer)	−20.70	−12.00	−56.75	1.024 × 10^−10^	163.314
(**b**) Supplementary preprocessing comparisons					
**Method**	**Mean SNR (dB)**	**Median SNR (dB)**	**SNR P10 (dB)**	**Median RMSE (T)**	**Median NRMSE over 101–1001 μs**
Wavelet-preprocessed input	−2.87	−3.51	−6.92	4.039 × 10^−11^	18.960
Median filtering (validation-selected; wavelet-preprocessed input)	0.49	−0.37	−2.93	2.869 × 10^−11^	6.073
VMD (validation-selected; wavelet-preprocessed input)	1.16	−0.08	−2.45	2.726 × 10^−11^	8.176

**Note:** Mean SNR is the arithmetic mean of per-trace SNR values in dB, not an aggregate SNR calculated by pooling amplitudes. P10 is the 10th percentile of per-trace SNR. RMSE is expressed in T, and NRMSE is dimensionless. All 1000 traces yielded finite SNR values for each listed pipeline. The archived PC-BiLSTMNet row represents transfer from a different training distribution and must not be interpreted as evidence of architecture-only superiority. These blind-test settings differ from the archived-benchmark settings in Table 1.

## Data Availability

A minimum reproducibility package, including the essential source code for synthetic-data generation, preprocessing, WL-CNN-BiLSTM implementation, training and evaluation, together with a small synthetic example dataset illustrating the input–target format and processing workflow, is provided as a Supplementary File. The supplied generation code can be used to generate additional synthetic samples according to the procedures described in this manuscript. The field AEM dataset is not publicly available because of confidentiality restrictions and is not included in the Supplementary File.

## Supplementary Materials

The following supporting information can be downloaded at: https://www.mdpi.com/article/10.3390/s26217021/s1, Table S1: Sensitivity of per-trace physical-domain reconstruction metrics to exclusion of the first receiver sample. Results use the saved predictions of the frozen layered-earth WL-CNN-BiLSTM configuration: (a) impulse-probability conditions corresponding to Table 6; (b) noise-stress conditions corresponding to Table 7b. Each condition contains the same 500 clean traces.

## Abbreviations

The following abbreviations are used in this manuscript:

AEM	Airborne Electromagnetic
CNN	Convolutional Neural Network
db4	Daubechies-4 (wavelet)
DWT	Discrete Wavelet Transform
GPU	Graphics Processing Unit
LSTM	Long Short-Term Memory
MAE	Mean Absolute Error
PCC	Pearson Correlation Coefficient
PC-BiLSTMNet	Parallel Convolution Bidirectional Long Short-Term Memory Network
ReLU	Rectified Linear Unit
RMSE	Root Mean Square Error
SimPEG	Simulation and Parameter Estimation in Geophysics
SNR	Signal-to-Noise Ratio
TEM	Transient Electromagnetic
VMD	Variational Mode Decomposition
WL-CNN-BiLSTM	Wavelet-enhanced Convolutional Neural Network–Bidirectional Long Short-Term Memory
AdamW	Adam with Decoupled Weight Decay
EMD	Empirical Mode Decomposition
IP	Induced Polarization
MSE	Mean Squared Error
ReduceLROnPlateau	Learning-Rate Reduction on Plateau Scheduler
SVD	Singular Value Decomposition
