# Supplementary material for: A Deep Learning-Based Denoising Method for Airborne Electromagnetic Data
Source: Sensors (Basel). 2026 Nov 6;26(21):7021. doi: 10.3390/s26217021 (PMC13766519; doi:10.3390/s26217021)
Supplement: Supplementary file 1 [file sensors-26-07021-s001.zip › sensors-4570711-supplementary.pdf]

Supplementary Table

Table S1. Sensitivity of per-trace physical-domain reconstruction metrics to exclusion of the first receiver sample. Results use the saved predictions of the frozen layered-earth WL-CNN-BiLSTM configuration: (a) impulse-probability conditions corresponding to Table 6; (b) noise-stress conditions corresponding to Table 7(b). Each condition contains the same 500 clean traces.

(a) Impulse probability

Condition	Receiver samples	Median SNR (dB)	SNR P10 (dB)	SNR P90 (dB)	Median RMSE (T)	Median NRMSE	Median NRMSE over 101–1001 μs
0%	101	6.0398	−44.9543	16.7408	2.293518×10^{-11}	0.4989	9.8860
0%	100	−6.2127	−40.0287	20.6722	3.740948×10^{-12}	2.0447	9.8860
5%	101	8.2675	−44.9544	19.4445	2.212253×10^{-11}	0.3860	9.9148
5%	100	−6.1438	−40.0301	20.7770	3.717059×10^{-12}	2.0286	9.9148
10%	101	7.7652	−44.9543	17.8379	2.238643×10^{-11}	0.4090	9.7368
10%	100	−5.8800	−40.0302	19.7239	3.720852×10^{-12}	1.9680	9.7368
20%	101	5.8072	−44.9541	18.5080	2.255546×10^{-11}	0.5124	10.5736
20%	100	−6.6604	−40.0148	16.8575	3.742348×10^{-12}	2.1529	10.5736
30%	101	6.9046	−44.9543	16.6101	2.255618×10^{-11}	0.4516	9.6433
30%	100	−6.5580	−40.0191	14.0164	3.797775×10^{-12}	2.1282	9.6433
40%	101	4.8007	−44.9542	15.5978	2.302228×10^{-11}	0.5755	10.6508
40%	100	−7.3768	−40.0392	13.9032	3.887307×10^{-12}	2.3380	10.6508

Note: The full evaluation contains 101 samples from 1 to 1001 μs. The diagnostic evaluation contains the remaining 100 samples from 11 to 1001 μs. The "Receiver samples" column identifies the interval used for SNR, RMSE, and the column labelled "Median NRMSE". Predictions were held fixed; no inference, retraining, filtering, or data generation was performed. Both reference and error energies were recomputed on each evaluation interval. P10 and P90 use linear interpolation across per-trace SNR values. No traces were removed. The final column reports median per-trace NRMSE over the fixed 101–1001 μs evaluation window, which contains 91 of the original 101 samples. This window is identical in both evaluations, so its NRMSE values remain unchanged. The specified window does not by itself identify a physical late-time regime. RMSE is in T; NRMSE is dimensionless. The full-interval metrics remain the primary results.

(b) Noise stress

Condition	Receiver samples	Median SNR (dB)	SNR P10 (dB)	SNR P90 (dB)	Median RMSE (T)	Median NRMSE	Median NRMSE over 101–1001 μs
Paired reference	101	7.2593	−44.9542	17.3515	2.249851×10^{-11}	0.4335	9.8900
Paired reference	100	−6.5637	−40.0251	14.7058	3.757389×10^{-12}	2.1291	9.8900
Gaussian scale 60%	101	6.5905	−44.9542	17.3309	2.272267×10^{-11}	0.4682	10.0092
Gaussian scale 60%	100	−6.4797	−40.0246	14.7265	3.752747×10^{-12}	2.1086	10.0092
Gaussian scale 75%	101	6.4901	−44.9542	16.6384	2.276958×10^{-11}	0.4737	9.8933
Gaussian scale 75%	100	−6.4491	−40.0243	14.8768	3.758989×10^{-12}	2.1011	9.8933
Gaussian scale 100%	101	6.5004	−44.9542	16.4319	2.275560×10^{-11}	0.4732	9.7798
Gaussian scale 100%	100	−6.4022	−40.0242	14.8824	3.797988×10^{-12}	2.0898	9.7798
Impulse peak scale 200%	101	1.6121	−44.9543	13.4408	2.341010×10^{-11}	0.8306	11.4468
Impulse peak scale 200%	100	−9.4228	−40.0243	13.4236	3.946160×10^{-12}	2.9590	11.4468
Impulse peak scale 250%	101	−0.9057	−44.9543	12.1426	2.349236×10^{-11}	1.1099	12.7430
Impulse peak scale 250%	100	−11.4393	−40.0237	10.9511	4.243284×10^{-12}	3.7323	12.7430
Impulse peak scale 300%	101	−2.6574	−44.9543	11.2517	2.350620×10^{-11}	1.3581	14.1090
Impulse peak scale 300%	100	−12.9067	−40.0228	10.1403	4.937364×10^{-12}	4.4191	14.1090
Impulse probability 40%	101	5.1974	−44.9541	16.5154	2.293230×10^{-11}	0.5497	10.0439
Impulse probability 40%	100	−7.2542	−40.0279	14.2935	3.815204×10^{-12}	2.3052	10.0439
Impulse probability 50%	101	4.6977	−44.9541	16.3233	2.291017×10^{-11}	0.5823	10.8134
Impulse probability 50%	100	−7.0500	−40.0155	13.3083	3.882754×10^{-12}	2.2516	10.8134
Added 50-Hz sinusoid	101	−1.5630	−44.9542	13.5618	2.341907×10^{-11}	1.1972	15.0044
Added 50-Hz sinusoid	100	−12.5605	−40.0009	9.6052	4.978401×10^{-12}	4.2465	15.0044

Condition	Receiver samples	Median SNR (dB)	SNR P10 (dB)	SNR P90 (dB)	Median RMSE (T)	Median NRMSE	Median NRMSE over 101–1001 μs
Added 1-kHz sinusoid	101	1.4779	−44.9537	13.6578	2.339200×10^{-11}	0.8435	11.4957
Added 1-kHz sinusoid	100	−9.5491	−40.0456	10.2076	4.133112×10^{-12}	3.0023	11.4957
Added 5-kHz sinusoid	101	2.6943	−44.9541	14.5153	2.335706×10^{-11}	0.7333	9.7666
Added 5-kHz sinusoid	100	−8.3517	−40.0253	14.5060	3.790927×10^{-12}	2.6158	9.7666
Gaussian 75% + impulse peak scale 200%	101	1.7331	−44.9542	13.6127	2.340674×10^{-11}	0.8191	11.4695
Gaussian 75% + impulse peak scale 200%	100	−9.3119	−40.0237	11.8516	3.988770×10^{-12}	2.9214	11.4695

Note: The full evaluation contains 101 samples from 1 to 1001 μs. The diagnostic evaluation contains the remaining 100 samples from 11 to 1001 μs. The "Receiver samples" column identifies the interval used for SNR, RMSE, and the column labelled "Median NRMSE". Predictions were held fixed; no inference, retraining, filtering, or data generation was performed. Both reference and error energies were recomputed on each evaluation interval. P10 and P90 use linear interpolation across per-trace SNR values. No traces were removed. The final column reports median per-trace NRMSE over the fixed 101–1001 μs evaluation window, which contains 91 of the original 101 samples. This window is identical in both evaluations, so its NRMSE values remain unchanged. The specified window does not by itself identify a physical late-time regime. RMSE is in T; NRMSE is dimensionless. The full-interval metrics remain the primary results.
